# A simple strategy guides the complex metabolic regulation in *Escherichia coli*

**DOI:** 10.1038/srep27660

**Published:** 2016-06-10

**Authors:** Giuseppe Facchetti

**Affiliations:** 1 Dept. Molecular and Statistical Physics, SISSA – International School for Advanced Studies, Trieste, Italy; 2 ICTP– International Centre of Theoretical Physics, Trieste, Italy

## Abstract

A way to decipher the complexity of the cellular metabolism is to study the effect of different external perturbations. Through an analysis over a sufficiently large set of gene knockouts and growing conditions, one aims to find a unifying principle that governs the metabolic regulation. For instance, it is known that the cessation of the microorganism proliferation after a gene deletion is only transient. However, we do not know the guiding principle that determines the partial or complete recovery of the growth rate, the corresponding redistribution of the metabolic fluxes and the possible different phenotypes. In spite of this large variety in the observed metabolic adjustments, we show that responses of *E. coli* to several different perturbations can always be derived from a sequence of greedy and myopic resilencings. This simple mechanism provides a detailed explanation for the experimental dynamics both at cellular (proliferation rate) and molecular level (^13^C-determined fluxes), also in case of appearance of multiple phenotypes. As additional support, we identified an example of a simple network motif that is capable of implementing this myopic greediness in the regulation of the metabolism.

The understanding of the ability of microorganisms to respond to various external perturbations represents an important but open question within Biology. A considerable effort has recently been devoted to studying the effect of different genetic and environmental changes, both experimentally and from a theoretical point of view, e.g. by measuring genes expression, ^13^C-based fluxes^1^,^2^,^3^,^4^ and by reconstructed genome-scale metabolic networks^5^,^6^,^7^. Through this perturbation analysis we hope to discover a global picture of the regulatory machinery that control the cellular metabolism. For example, it has been found that microorganisms like *Escherichia coli* respond to a gene knockout by arresting their proliferation and by activating a large set of alternative pathways, the so called latent pathways^8^. Nevertheless, this “survival” response is only transient because, by re-routing the activation of the metabolic reactions, the microorganism is able to rescue its growing capability^9^,^10^. However, depending on the perturbation and on the nutrients availability, this proliferation recovery can be complete or only partial. An analysis of the published results considered in this work shows that, differently from what is usually assumed^11^, there is a significant fraction of cases (about 20%, see Supplementary Fig. S1 and ref. 9) in which the maximal value of the growth rate is not achieved. Therefore, although new experimental results from specific perturbations always provide useful and valuable information, it is also worth to consider the ensemble of the available knowledge and try to find a unifying explanation. Why are there perturbations for which the microorganism is unable to reach the maximal growth rate? Why after some knockouts does the microorganism adopt that redistribution of the active pathways even if it does not provide an optimal recovery of the metabolic function? Different definitions of optimality, like for instance Pareto optimality^12^, do not provide a description of the regulatory mechanism that guides each step of the response dynamics of the microorganism. On the other hand, in the computational methods available in the current literature, dynamics and regulation are introduced by imposing an external time-dependent process (for example the consumption of a limited amount of nutrients or the intracellular crowding^13^) or by setting extra constraints and parameters, which are obtained by additional experimental measurements^11^,^14^,^15^,^16^. Therefore, none of these methods provides a unifying and simple explanation for the complex metabolic response.

In order to find such a basic interpretation, our analysis consists of four steps: (I) from a general biological *rationale* that does not make use of any specific information from the available experiments, we formulated a simple hypothesis about a possible unifying principle; (II) we validated the proposed hypothesis by a stoichiometric reaction model: without any tuning of the parameter to fit the experimental data, we tested whether experimental results can be reproduced by this hypothesis, both as growth rate recovery and as fluxes redistribution; (III) once it has passed this validation, the criterion is used to predict and to derive an explanation for some interesting cases of metabolic responses; (IV) finally, we showed how a possible motif in the regulatory network can implement the proposed principle at the molecular level.

## Results

### From general biological considerations to the greedy hypothesis

Our starting point is the following *rationale*: as already mentioned, genetic and environmental perturbations cause a transient activation of the non-essential metabolic pathways that are progressively silenced as the microorganism adapts to the new condition^17^. Indeed, it is known that a resilencing after a knockout can lead to the recovery of some cellular functions such as the growth rate^10^. In a microorganism, the resilencing of a reaction is achieved through different feedback/feedforward mechanisms such as, for instance, down-regulation of gene expression, the allosteric effect of a metabolite, deactivation of the enzyme by phosphorylation or dephosphorylation. Clearly, all these mechanisms rely only on the information that is available to the cell at the time of the resilencing (a myopic view). Indeed, it is reasonable to assume that the cell does not know the final point of the entire adaptation, neither as growth rate nor as activated/silenced pathways. Based on this simple and plausible reasoning, we hypothesized the following greedy heuristic: among all the activated reactions, the regulatory machinery of the cell had evolved in order to preferentially select the resilencing that triggers a high (greedy) instantaneous (myopic) proliferation advantage.

### Validation of the greedy hypothesis

To validate this hypothesis, we tested how a recursive greedy resilencing adjusts the central metabolism of *E. coli* after a wide set of different perturbations (see Supplementary Fig. S2 and Table S1). Starting from a Flux Balance Analysis (FBA) method recently developed^18^, we built a procedure that recursively and stochastically adopts a greedy choice among all possible resilencings: *the higher the induced recovery of the growth rate*, *the higher the probability for the cell to adopt that resilencing*. In particular, the following equation has been used for the estimation of probability of resilencing reaction *k*:


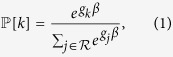


where *g*_*j*_ is the short-term recovery of the growth rate induced by resilencing reaction *j* and obtained by MOMA^6^, *β* is the unique model parameter that describes the amount of randomness in the simulation and 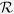 is the set of all active reactions, i.e. the set of all possible resilencings (see Equations S6 and S7 in the Supplementary text for more details). From the recursive use of this resilencing-MOMA procedure, we obtained a Markov Chain whose evolution generates a sequence of metabolic adjustments (Supplementary Fig. S3). We called the method GRAM: Greedy Resilencing in the Adjustment of Metabolism. It is worth noting that the method does not use any additional constraints derived from transcriptional and regulatory information, and it does not introduce any artificial bias on the choice of the resilencings. The entire dynamics are merely determined by the greedy criterion and by the given stoichiometry of the metabolic network. Moreover, because of the absence of any fitting procedure for the parameter *β* of the model (see Supplementary Fig. S4 for the choice of *β* = 200 h) and because of the complete independence of the construction of the method from the specific experimental results it is asked to describe, we believe that the comparison between simulations and experimental data represents a stringent test on the validity of our hypothesis.

**Figure 1:**
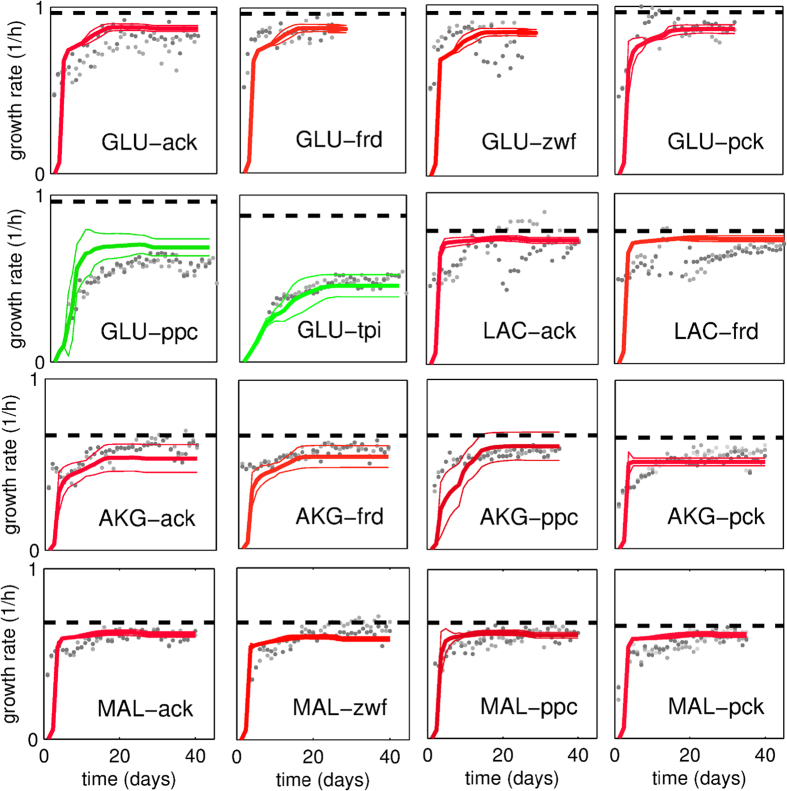
Dynamics of the proliferation recovery of *E. coli* for different experimental conditions. Each panel refers to the indicated pair carbon source-knockout and reports the results obtained with our criterion and the experimental data from ref. 9. *Legend*. AKG: *α*-ketoglutarate, GLU: glucose, LAC: lactate, MAL: malate, SUC: succinate. Curves represent the results from GRAM (thick and thin lines: average and average ± standard deviation). Red: complete or almost complete recovery (>90%); green: partial recovery (<90%). Gray dots: experimental values. Dashed black line: maximal growth rate (FBA biomass optimization). The SUC-*tpi* case has not been reported because it is lethal, i.e. a constant zero growth rate for both experiments and model.

We considered 17 different combinations of single carbon sources media with gene knockouts on *E. coli*, namely glucose, *α*-ketoglutarate, lactate, malate and succinate as carbon source and acetate kinase (*ack*), fumarate reductase (*frd*), glucose-6-phosphate dehydrogenase (*zwf*), phosphoenolpyruvate carboxylase (*ppc*), phophoenolpyruvate carboxykinase (*pck*) and triose-phosphate isomerase (*tpi*) as deleted genes (see Supplementary Table S1 for the complete list). For each condition we simulated how the proposed greedy hypothesis would describe the metabolic regulation. The plots in Fig. 1 report the computed recoveries of the growth rate and the comparison with experimental data from ref. 9. For all substrates and knockouts, the entire experimental dynamics of the recovery of the growth rate follows very closely the trajectory provided by our greedy hypothesis. Of particular interest is the case of glucose as the carbon source: our criterion explains the fact that both Δ*tpi* and Δ*ppc* show a slower and only partial recovery of the growth rate, i.e. they do not reach the maximal value predicted through the classical biomass optimization (green colour). These results suggested then a general validity of the greedy hypothesis regardless of the knockout and growing condition.

**Figure 2:**
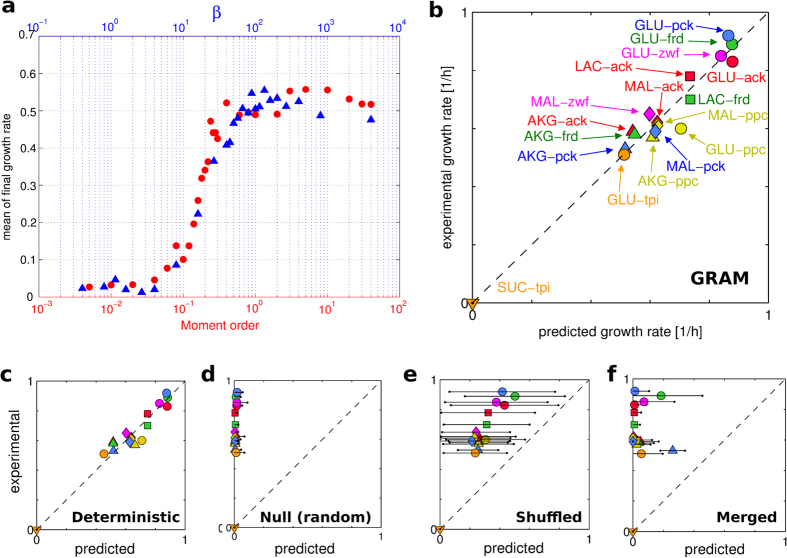
Tests on the importance of greediness. Comparison of the final growth rate obtained from some variation of greediness. (**a**) GLU-Δ*tpi*: results by Boltzmann equation (1) (blue ▴, top *x* axis) and by moment equation (2) (red ⦁, bottom *x* axis) at different value of *β* and *m*, respectively. (**b**) Results from GRAM; RMSD of the mean = 0.0616. (**c**,**d**) Results from deterministic and random (null) models; RMSD of the mean = 0.0783 and 1.94 respectively. (**e**,**f**) Results from shuffled and merged sequence of the resilencings obtained from the standard GRAM procedure; RMSD of the mean = 0.752 and 1.57, respectively). Growth rates in h^−1^. Black horizontal lines: error bar (standard deviation). Color code for knockouts: red = *ack*, green = *frd*, violet = *zwf*, yellow = *ppc*, blue = *pck*, orange = *tpi*. Legend for carbon sources: *α*-ketoglutarate (AKG, Δ), glucose (GLU, ⚪), lactate (LAC, ◽), malate (MAL, ♢) and succinate (SUC, 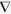).

It is worth noting that also in the case of high recovery, simulated growth rate does not reach exactly the maximal value suggested by classical FBA. Part of this small discrepancy might be due to the “calibration” of the nutrients availability (see Supplementary Fig. S2). However, we must also consider that, like for any optimization algorithm that differs from Linear Programming, there is no proof and guarantee that the proposed method should reach precisely the optimal solution given by the growth rate maximization. Indeed, consistent with our predictions, the experimental data also show that a truly complete recovery is not reached every time (see also Fig. 2b).

To further validate the greedy principle, we verified that the mathematical expression (1) used for greediness quantification was not stringent: as shown in Fig. 2a, the results indeed did not change when the *m*-th moment expression


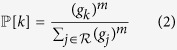


was used instead of the Boltzmann equation (1). Furthermore, the comparison with the FBA heuristics that are normally used showed our approach performed better, see Supplementary Fig. S5. Finally, we looked at how a deviation from greediness affects the quality of the results. In particular we tested a very greedy choice (purely deterministic, i.e. the cell always adopts the most convenient resilencing), the hypothesis of a random choice and the effect of shuffling or merging the sequence of the greedy resilencing we have identified. We ran each of these modified procedures for all the 17 experimental conditions. As shown in Fig. 2b–f, except for the pure deterministic version which still follows the greedy criterion, for all other deviations the agreement with the experimental data was significantly reduced or even lost. Therefore, also these tests supported our hypothesis.

**Figure 3:**
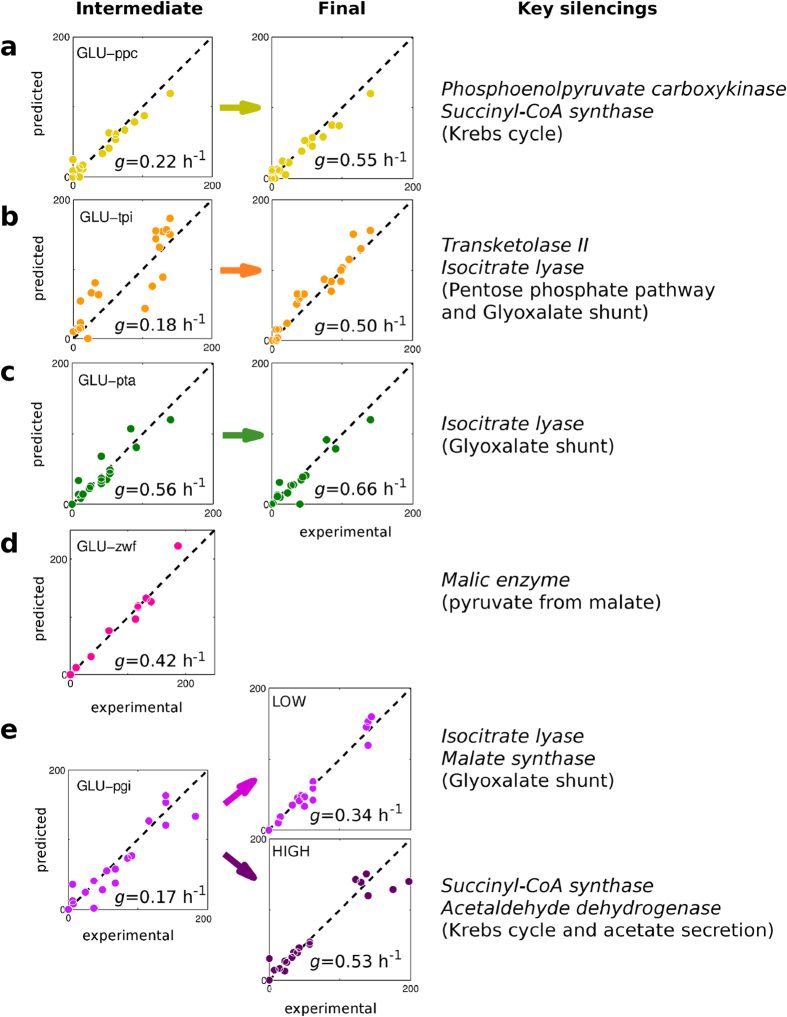
Comparison with ^13^C-labelling metabolic fluxes in glucose (from refs 19,20). Experimental fluxes are compared with the flux calculated with the greedy hypothesis for intermediate and final steps of the adaptation for different knock-outs. The intermediate states along the trajectory have been chosen based on the growth rate of the corresponding ^13^C-labelling experiments used for the validation. Value of this growth rate is reported in each panel. (**a**) GLU-*ppc* (40% of recovery with respect to the final growth rate); (**b**) GLU-*tpi* (36% of recovery with respect to the final growth rate); (**c**) GLU-*pta* (84% of recovery with respect to the final growth rate); (**d**) GLU-*zwf*, only experimental data for intermediate steps is available (48% of recovery with respect to the simulated end-point); (**e**) GLU-*pgi* (50% of recovery with respect to the final growth rate). In the latter case, the end-points for two different phenotypes are reported (denoted HIGH and LOW). For statistics and comparison with other methods see Supplementary Table S2. The last column reports the key silenced reactions (and pathways) predicted by GRAM and confirmed by the experimental masurements.

So far, we found that this greedy regulation of the metabolism appears to be a good principle for explaining the macroscopic evidence of the growth rate recovery. This led us to the question about the re-routing of the metabolic pathways. Can the redistribution of the reaction fluxes be explained by the greedy hypothesis? For this purpose we looked into the details of the dynamics by identifying which reactions have been resilenced/activated along the adjustment and comparing this result with the ^13^C-determined fluxes at the intermediate steps and at the final steps of the adjustment in glucose for five different knockouts, namely *zwf*, *ppc* and *tpi*, plus phosphate transacetylase *pta* and phosphoglucose isomerase *pgi*^19^,^20^. The results are displayed in Fig. 3 for intermediate and final points, together with the key resilencings: all these predicted resilencings were consistent with available ^13^C-labelling experimental data. Therefore, for each knockout and regardless to which intermediate state along the trajectory has been used for the validation (validation points have a percentage of recovery that varies from 36% to 84%), the observed flux re-routing can be well described by our hypothesis. A comparison with other FBA methods is reported in Supplementary Table S2 and confirms the better performance of GRAM.

As indicated in Fig. 3e a significant example is the *pgi* knockout. It has been shown that this mutant is characterized by a reduction of the growth rate to approximately 0.3 h^−1^ and by a re-routing of the fluxes toward the pentose phosphate pathway^21^,^22^. However, in the experiments reported in ref. 19 a second phenotype with high growth rate (0.53 h^−1^) has been identified. Measurements by ^13^C-labelling indicated that the difference between these two phenotypes is due to the use (and non-use, respectively) of glyoxalate shunt and of acetate secretion. Surprisingly, these two possible growth rates were obtained also in our trajectories: 0.31 and 0.60 h^−1^ with about 70% and 20% probability, respectively. As indicated by Fig. 3e, computed fluxes are consistent with ^13^C-measurements for both phenotypes (see Supplementary Fig. S7 for a sketch of the activated and silenced pathways in the two phenotypes). This agreement in the flux redistribution concerned also the adjustment of redox balance of the cell, i.e. the production or consumption of the NAD/NADH (see “Mutations” in Supporting Results). Therefore, even in the complex case of appearance of multiple phenotypes, the proposed principle is still able to explain the observed adjustments.

After having validated the hypothesis in terms of cellular growth rate and pathways re-routing, we looked at the importance of the temporal order in the regulation of the metabolism and how this order is captured by the greedy criterion. As reported above in Fig. 2e, an indirect proof was given by the loss of the prediction power when shuffling the sequence of resilencing: by altering the order given by greediness, the cell becomes unable to correctly rescue its metabolic functions. Indeed, although some reactions can be stopped in a large time window, there are crucial reactions for which the temporal order must be preserved (see Supplementary Fig. S8). The two examples about the resilencing of transketolase in GLU-*tpi* and about the resilencing of membrane-bound NADPH transhydrogenase in GLU-*pgi* are described in the next paragraph.

### Explanations from the greedy hypothesis

**Figure 4:**
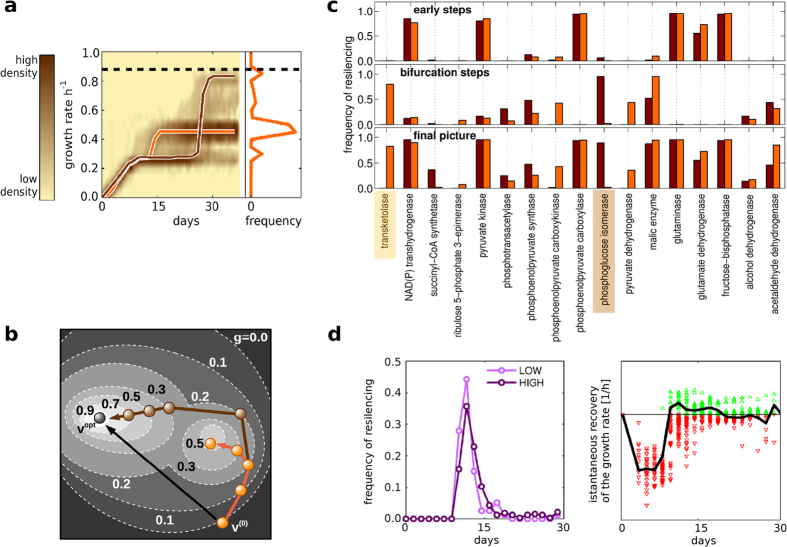
Greediness provides explanation for sub-optimality and timing of the adjustments. (**a**) GLU-*tpi* condition: density plot of the simulated time-recovery of the growth rate; the histogram at the right reports the frequency of the growth rates at the end-point. Two representative trajectories are shown explicitly: orange line refers to a trajectory with low final growth rate (0.48 h^−1^), brown line with high final growth rate (0.82 h^−1^). Same colours used in (**b**,**c**). (**b**) Interpretation of the dynamics of panel (**a**) as trajectories of a particle in a energy landscape where energy *H* = −*g*. (**c**) Frequency of resilencing of critical reactions in the two GLU-*tpi* trajectories at the early, bifurcation and final steps (normalization with respect to the maximal value). Although at the early steps the two trajectories have similar flux redistributions, a crucial intermediate resilencing (transketolase II is the greedy option, phosphoglucose isomerase is the non-greedy one) leads to different end-points. (**d**) *Left*: in GLU-*pgi* simulation, resilencing of membrane NADPH transhydrogenase always occurs after 7 days (for LOW and HIGH phenotype, see Fig. 3e). *Right*: dots represent the instantaneous effect on the proliferation recovery depending on the resilencing time of membrane NADPH transhydrogenase (green Δ: positive recovery; red 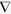: negative recovery). Each dot refers to a time point of a single trajectory. Black curve represents the average.

With the detailed description provided by GRAM about the flux redistribution, we have now the possibility to decipher the mechanism that controls the metabolism. Our aim, indeed, is not to reproduce data that are already known from previous experiments, rather we want to provide a possible explanation for why such results have been observed. With this purpose, we present two significant examples in Fig. 4.

As seen before, Δ*tpi* is a very interesting perturbation after which the microorganism reaches only a partial recovery of the growth rate up to 0.50 ± 0.02 h^−1 ^ (ref. 20), which is only half of the optimal value 0.87 h^−1^ obtained from the biomass maximization in FBA. Both GRAM results and ^13^C-data indicate that the microorganism stops the pentose phosphate pathway and uses glycolysis as the main catabolic route^19^. Therefore, we would like to find the crucial step of the adjustment that explains this sub-optimal strategy and by which entity it fits into the greedy hypothesis. The trajectories of our model show a dominant adaptation strategy with a final growth rate equal to 0.48 h^−1^ (see orange line in Fig. 4a). Nevertheless, with a much lower probability (only 15%), the simulations show also a second set of trajectories which instead reach a growth rate of about 0.82 h^−1^ (brown line in Fig. 4a). The bifurcation between these two dynamics occurs after about 2 weeks. By looking at the resilencing adopted at these intermediate steps, we found that transketolase II on the pentose phosphate pathway is always resilenced in the most frequent trajectories that reach low proliferation (orange bars in Fig. 4b), whereas it is never resilenced in the rare trajectories that end with high proliferation and for which phosphoglucose isomerase is instead stopped (brown bars in Fig. 4b). Moreover, we can also see that the resilencing of transketolase II provides a short-term recovery of the biomass production up to 0.45 h^−1^ (see jump of orange curve after 15 days in Fig. 4a) which is higher than the alternative resilencing of phosphoglucose isomerase in the glycolysis pathway (up to 0.25 h^−1^, see brown curve in Fig. 4a after 15 days). Therefore, given these two possible resilencings, our criterion suggests that the microorganism would preferably choose the resilencing which assures a higher short-term recovery, i.e. it stops transketolase II in the pentose phosphate pathway and keeps glycolysis as the active metabolic route. Unfortunately, since the *tpi* knockout reduces the global long-term efficacy of glycolysis (unknown to the myopic cell at this bifurcation point), the final outcome is a phenotype with low proliferation rate. Figure 4c shows an interpretation in terms of an energy landscape, where the moving particle is attracted and trapped in a local minimum.

The fact that the highly proliferating phenotype has not been observed can then be interpreted as a confirmation of the existence of the greedy strategy (i.e. the low probability for the resilencing of phosphoglucose isomerase): if the cells do not follow the greedy criterion, it would have been easier to find this high proliferation phenotype. Moreover, as seen in the trajectories in Fig. 4a, there is a long transient dormant state in the non-greedy dynamics (brown curve) that might further reduce the experimental detectability of the high proliferation phenotype: indeed, after the silencing of phosphoglucose isomerase, the growth rate remains at a low value (0.25 h^−1^) for about 10 days making these cells unable to compete for the common nutrients against the greedy cells that are transiently proliferating faster (0.45 h^−1^, orange curve).

Concerning the resilencing time, Fig. 4b shows that in this mutant some reactions can be resilenced almost at any time of the dynamics (see for example pyruvate kinase, glutaminase and fructose-biphosphatase) whereas reactions which are crucial for the bifurcation (transketolase II and phosphoglucose isomerase) must be stopped only at the intermediate stage of the dynamics.

Finally, the use of GRAM provided also an interesting explanation for the apparent inconsistency regarding the resilencing of the membrane-bound NADPH transhydrogenase in the GLU-*pgi* condition. Indeed, although a mutation that stops this reaction has been found in the adaptation of GLU-*pgi*^23^, it is not known why the insertion of the same mutation (i.e. at time zero) showed a clear disadvantage for the microorganism: the final growth rate was only 0.1 h^−1^, instead of 0.3 or 0.6 h^−1 ^ (ref. 23). The exact time of this adaptation step is not known. In our trajectories it was instead possible to see that membrane-bound NADPH transhydrogenase was silenced approximately after 11 days and never before 7 days (Fig. 4d). Moreover, the same results showed that the effect of this resilencing was always negative during the first week (red points in Fig. 4d). Therefore, the greedy interpretation suggests that, only once other adjustments have already been adopted, does the regulation of the NADPH metabolism become convenient. This is the reason why a forced stop of this reaction at time zero was not beneficial for the microorganism.

### An example of a greedy regulatory motif

As mentioned, it is known that a cell can resilence a reaction through different feedback/feedforward mechanisms that down-regulate gene expression, have an allosteric effect on the enzymatic activity, or phosphorylate/dephosphorylate a protein. Therefore, the following question might arise: how can these mechanisms implement the proposed greedy strategy in a cell? To answer this question, we showed how a simple negative feedback motif, which is common in the regulatory networks, is capable of performing a greedy choice. Clearly, the set of feedbacks we present here is just an example and other alternative motifs with greedy behaviour may exist.

We used this motif in a minimal model. Because of its simplicity, local information coincides with global information, so that a greedy strategy always leads to the optimal point. However, we first focused our study on this toy network because it allows to easily identify the key biological features that are essential for the implementation of greediness.

**Figure 5:**
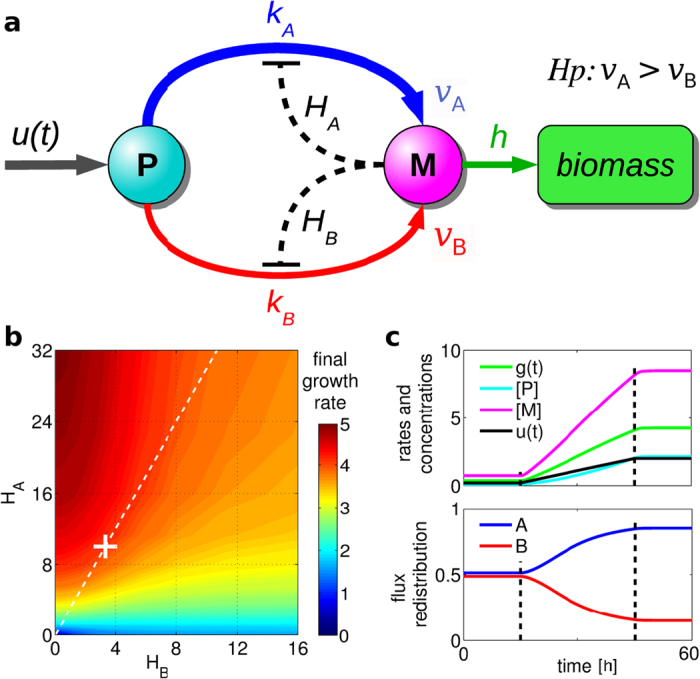
An example of a possible greedy regulatory motif. (**a**) Sketch of the network: the metabolite *M* has an inhibitory effect on the two enzymes that convert a molecule of the precursor *P* into *ν*_*A*_ = 3 or *ν*_*B*_ = 1 molecules of *M* by reaction *A* or by reaction *B*, respectively. The two inhibitions are described through quadratic Hill equations with different constants *H*_*A*_ and *H*_*B*_, see equation (3). (**b**) Effect on the final growth rate of the Hill constants *H*_*A*_ and *H*_*B*_ (all other parameter values in Fig. S10). White dotted line indicates the set of points such that *H*_*A*_/*H*_*B*_ = *g*_*B*_/*g*_*A*_ (where *g*_*A*_ and *g*_*B*_ are the growth rates after resilencing reaction *A* and *B*, respectively – see Supplementary Fig. S9). White cross corresponds to the values used for the simulation in panels c, namely *H*_*A*_ = *g*_*B*_/*h* = 10 and *H*_*B*_ = *g*_*A*_/*h* = 3.3. (**c**) Above: Simulated dynamics of the growth rate *g*(*t*) = *h*[*M*] (green) and of the concentration of the two compounds, *P* (cyano) and *M* (magenta), for a given precursor availability *u*(*t*) as input (black). Below: Redistribution of the fluxes between the two parallel reactions *A* and *B* (same simulation as above). More simulations in Supplementary Fig. S9. An example of network in which greediness leads to a suboptimal end-point is presented in Supplementary Fig. S10.

The sketch of the simple motif is reported in Fig. 5a: two parallel pathways, here condensed in reactions *A* and *B*, convert precursor *P* in to metabolite *M* with stoichiometry 1:*ν*_*A*_ and 1:*ν*_*B*_, respectively (i.e. 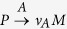 and 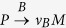). Without loss of generality we assumed reaction *A* is more efficient than reaction *B* (i.e. *ν*_*A*_ > *ν*_*B*_). Since *M* is used for the biomass production, this implies also that the growth rate recovery induced by resilencing reaction *A* is smaller that the growth rate recovery induced by the resilencing of reaction *B* (*g*_*A*_ < *g*_*B*_, see Supplementary Fig. S9). The two negative feedbacks of the metabolite *M* on reactions *A* and *B* (e.g. allosteric effect) have been described through classical Hill equations with constants *H*_*A*_ and *H*_*B*_, respectively. This led to the following set of equations:


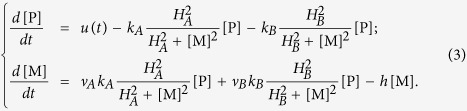


The growth rate is then given by *g*(*t*) = *h*[M]. We considered the case when a previous step in the regulation dynamics has caused an increase of the availability *u*(*t*) of the precursor *P*. Given such a input, we analysed how the two feedbacks may affect the response of the systems to this change. Figure 5b reports the biomass production obtained by running the simulations with different values of *H*_*A*_ and *H*_*B*_. This result shows that, in order to induce an higher increase of the growth rate, it is sufficient that evolution had shaped the inhibitory effects of *M* by increasing *H*_*A*_ and/or decreasing *H*_*B*_ such that *H*_*A*_ > *H*_*B*_. We would like to point out that, as mentioned above, in such a simple network the induced short-term increase of the growth rate coincides with a global advantage: this is the reason why this system reaches high biomass production.

It is also interesting to estimate the probability of resilencing reaction *A* through its effective kinetic parameter in equation (3), i.e. 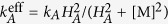. Since the resilencing of reaction *A* gives *g*_*A*_ = *h*[M], this leads to:


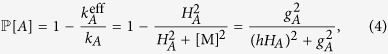


which, by choosing *H*_*A*_ such that *hH*_*A*_ = *g*_*B*_, is identical to the second-moment formula (2) that we already proved to describe well the metabolic regulation (see Fig. 2a from which we can also see that 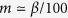, i.e. *m* = 2). Indeed, simulations of the network motif (3) with *H*_*A*_ = *g*_*B*_/*h* and *H*_*B*_ = *g*_*A*_/*h* reproduces the resilencing of reaction *B*, coherently with our greedy criterion (see white cross in Fig. 5b and corresponding dynamics in Fig. 5c; more simulations in Supplementary Fig. S9). This indicates that evolution could have modulated these values and thus shaped the metabolic regulation in order to implement the greedy strategy.

As mentioned at the beginning of this section, with such a minimal model it was not possible to differentiate local from global information, i.e. the short-term advantage in the growth rate recovery was also a long-term advantage. Therefore, in order to have a greedy and myopic dynamics that ends in a suboptimal point, a more complex network has been built. Supplementary section “An example of network with suboptimal end-point” presents an example of such a network and confirms that the proposed regulatory motif based on negative feedbacks still guarantees the implementation of the greedy strategy also in case of a partial recovery of the growth rate.

## Discussion

Cases of sub-optimal adjustment of the metabolism have suggested a myopic strategy as a guide for the microbial response to external perturbations: in our hypothesis this strategy is the myopic greedy resilencing. The description we obtained for the considered wide range of different gene deletions and different carbon sources suggests that this principle is fundamental. Indeed, our criterion provides an explanation for important experimental features both at the macroscopic level and at the molecular level also for non-trivial metabolic responses such as the appearance of multiple phenotypes (Figs 1, 2, 3, 4). It is in fact reasonable to think of the regulatory machine as having already been shaped by evolution in order to favour the recovery of the growth rate. However, it is unlikely that evolution has led to a system capable of an optimal recovery from all possible external perturbations. Similarly, in the case of bacterial chemotaxis the unavoidable conflicting requirements that come from different and incompatible chemoattractant profiles have shaped the signalling pathway towards a compromised chemotaxis response^24^. In this perspective, the proposed criterion of a greedy and myopic regulation appears to be the most reasonable and natural solution found by evolution.

The analogy with the energy minimization problem over a corrugated landscape is also straightforward. Considering the growth rate as the opposite of a Hamiltonian, a metabolic adjustment can be viewed as the dynamics of a particle that is moving toward the minimal energy (see Materials and Methods and Supplementary Figs S11 and S12 for more details). In this perspective, the regulatory machine might be seen as an algorithm that searches for the global minimum. In fact, it is well known that, depending on the initial point and on the complexity of the energy landscape, even the best available algorithm cannot guarantee to find the optimal solution. Because of this, it is reasonable to expect that also the microorganism may end in a local minimum (as seen for GLU-*tpi* and GLU-*pgi*).

Moreover, we have presented an example of a simple regulatory motif which might be used by the cell to adopt the greedy strategy (Figs 5 and S10). While doing this, we showed how the description provided by GRAM is equivalent to the description derived from classical Hill equations (see equation (4)). Therefore, although the greedy hypothesis has been formulated through a very different and general approach, the resulting GRAM method is consistent with the standard mathematical formalism used for modelling cell regulation. All these three similarities with consolidated approaches might be considered as additional support to the plausibility of our interpretation as the basic and fundamental principle.

We would also like to underline that, the proposed method does not require any pre-existing information about the regulations. Since the entire description is based only on the stoichiometry of the reaction network, the proposed mechanism establishes a new functional link between regulation and stoichiometry of the metabolism. Therefore, as pointed out by the case of multiple phenotypes appearance we had considered in the validation step, our interpretation might also contribute for a better understanding of the genotype-phenotype correlation. For the same reason, our method introduces a new use of FBA which may enlarge the spectrum of applications of this computational tool inside Systems Biology.

## Materials and Method

The reconstructed stoichiometric network of the central metabolism of *E. coli*^25^ has been extended to incorporate the methylglyoxal pathway. Supplementary Fig. S2 reports a sketch and the main features of this network. Moreover, after having verified that the carbon source is the only limiting factor for the biomass production, the upper-bound of its corresponding exchange reaction has been rescaled to the measured Supply Uptake Rate (SUR)^9^. Values are plotted in panel C of Supplementary Fig. S2. This constraint was necessary in order to reproduce the experimental growing conditions and to provide the quantitative and absolute scaling of the computed growth rate with respect to the experimental values. The same procedure had been adopted in ref. 9. However, in order to avoid any influence on the redox balance of the metabolism, we did not set any constraint on the Oxygen Uptake Rate (OUR).

GRAM represents an improvement of what has been reported in ref. 18: the current version provides a more useful and necessary biological interpretation of the phenomenon we want to describe and explain. For completeness, we reported the entire formalism in the Supplementary Information. The code for GRAM and the model of equation (3) have been implemented in MATLAB 2013R using ILOG-IBM CPLEX 12.1 (under academic licence) for solving all linear and quadratic optimization problems. All results from GRAM are the average over a set of 500 independent simulations using β = 200 h.

## Additional Information

**How to cite this article**: Facchetti, G. A simple strategy guides the complex metabolic regulation in *Escherichia coli*. *Sci. Rep*. **6**, 27660; doi: 10.1038/srep27660 (2016).

## Supplementary Material

Supplementary Information
