# Supplementary material for: A simple strategy guides the complex metabolic regulation in Escherichia coli
Source: Sci Rep. 2016 Jun 10;6:27660. doi: 10.1038/srep27660 (PMC4901314; doi:10.1038/srep27660)
Supplement: Supplementary Information [file srep27660-s1.pdf]

A simple strategy guides the complex metabolic regulation in *Escherichia coli*: SUPPLEMENTARY INFORMATION

Giuseppe Facchetti[1,2,*]

[1]Dept. Molecular and Statistical Physics, SISSA – International School for Advanced Studies, Trieste, Italy

[2]ICTP – International Centre of Theoretical Physics, Trieste, Italy

[*]current email address: giuseppe.facchetti@jic.ac.uk

1 METHOD

1.1 Greedy Resilencing in the Adaptation of Metabolism

Notations. Within the FBA framework, a metabolic network is represented by an $m \times r$ stoichiometric matrix $\mathbf{S}$ (m and r being the number of metabolites and of reactions, respectively) and by a vector $\mathbf{v} \in \mathbb{R}^r$ of reaction fluxes. For each flux, a lower and an upper bound is given, denoted by the vectors $\mathbf{L}$ and $\mathbf{U}$ respectively; $\mathbf{L}$ and $\mathbf{U}$ are related not only to the maximal concentration of the enzymes (expression of the coding genes and alterations due to knockouts or mutations) and to their activation (biochemical or allosteric), but also to the medium in which the microorganism is growing (nutrient inflows are among the fluxes of the metabolic network). Together with the steady state assumption ($\mathbf{Sv} = \mathbf{0}$), these constraints define the polytope

$$W(\mathbf{L}, \mathbf{U}) = \{\mathbf{v} \ \ : \ \ \mathbf{Sv} = \mathbf{0}, \ \ \mathbf{L} \leq \mathbf{v} \leq \mathbf{U}\},$$

here denoted as $W(\mathbf{L}, \mathbf{U})$ because it is viewed as a function of the lower and upper bounds which may change over time. Indeed, some components of $\mathbf{L}$ and/or of $\mathbf{U}$ can be set to zero following the silencing of the corresponding enzymes/genes or following the removal of some nutrients (whereas the stoichiometric matrix $\mathbf{S}$ does not change when a perturbation occurs). Therefore, in order to describe the long-term adjustment of the metabolism, we use a time-discrete dynamics represented by the sequence

$$\{[\mathbf{v}^{(s)}, \mathbf{L}^{(s)}, \mathbf{U}^{(s)}]\}_{s=0,1,2,\ldots},$$

where $\mathbf{v}^{(s)}$, $\mathbf{L}^{(s)}$ and $\mathbf{U}^{(s)}$ denote the reaction fluxes, the lower and upper bounds at step s, respectively. Each element of the sequence is characterized by the growth rate $g^{(s)} = v^{(s)}_{\text{biomass}}$.

The starting point $[\mathbf{v}^{(0)}, \mathbf{L}^{(0)}, \mathbf{U}^{(0)}]$. The experiments we consider in this paper are performed following a common laboratory protocol for adaptation assays in microorganisms [6, 4, 7, 8]. These experiments start with overnight growth in Luria-Bertani rich medium; at the beginning of the second day, the rich medium is substituted with a single carbon source minimal medium. To describe these experiments in our model, we first calculate the unperturbed fluxes (wild-type strain in rich medium) by a variant of the so called *parsimonious* FBA (pFBA) [14]. The maximization of the biomass component of the fluxes on the initial polytope $W(\mathbf{L}, \mathbf{U})$, i.e.

$$\mathbf{v}^{\text{wt}} = \arg \max_{\mathbf{v} \in W(\mathbf{L}, \mathbf{U})} v_{\text{biomass}}, \tag{S1}$$

provides the value of the maximal growth rate ($g^{\max} = v^{\text{wt}}_{\text{biomass}}$). Because of the non-uniqueness of the solution of this optimization problem, pFBA selects the reactions fluxes that have the least enzymatic cost (i.e. the minimal sum of the fluxes) while guaranteeing the maximal biomass. This is achieved through a second optimization:

$$\mathbf{v}^{\text{pFBA}} = \arg \min_{\substack{\mathbf{v} \in W(\mathbf{L}, \mathbf{U}) \\ v_{\text{biomass}} = g^{\max}}} \sum_{i=1}^{r} |v_i|. \tag{S2}$$

The knockout of reaction k in a mutant is introduced by setting $L_k = U_k = 0$. Similarly, the switch to the minimal medium is described by setting to zero the upper-bounds of all the nutrient uptakes, except for the carbon source used in the minimal medium and the other essential chemicals (oxygen, phosphate, ammonia, water, etc.). The negative lower bounds are not changed since the secretion of substances (for instance ethanol, acetate) is still possible in the minimal medium. At $s = 0$, the modified lower and upper bounds are denoted $\mathbf{L}^{(0)}$ and $\mathbf{U}^{(0)}$. The initial effect of the perturbation is modelled by MOMA, which has been proven to provide a reliable description of the short-term metabolic adjustment [15, 16]. The vector of fluxes after these perturbations is then:

$$\mathbf{v}^{(0)} = \arg \min_{\mathbf{v} \in W(\mathbf{L}^{(0)}, \mathbf{U}^{(0)})} \|\mathbf{v} - \mathbf{v}^{\text{pFBA}}\|_2, \tag{S3}$$

where $\| \cdot \|_2$ denotes the L^2-norm. In Fig. S3A, this step is described by the projection of the unperturbed point $\mathbf{v}^{\text{pFBA}}$ (blue) to the point $\mathbf{v}^{(0)}$ (red): the corresponding flux distribution, characterized by the growth rate $g^{(0)} = v^{(0)}_{\text{biomass}}$, is the starting point of our dynamics. Clearly, depending on the specific experiments one aims to reproduce, other possible definitions of the starting point can be adopted.

Given the polytope $W(\mathbf{L}^{(0)}, \mathbf{U}^{(0)})$, using Eq. (S1) and (S2) it is also possible to recalculate the optimal solution given by pFBA, and denote it $\mathbf{v}^{\text{opt}}$, with the corresponding growth rate g^{opt} ($\leq g^{\max}$ by construction, see Fig. S3A).

Resilencing probabilities at each step. As described in the main text, it is known that the recovery of the growth rate is accompanied by the resilencing of the activated non-essential reactions [13, 10, 11]. Of course, processes like the diffusion of a metabolite across the membrane cannot be regulated: such fluxes are then excluded from the set of possible resilencings. The first key point of GRAM is then the *iterative resilencing*: one by one, the active reactions are progressively silenced and the corresponding *short-term* effect is calculated through MOMA. For simplicity, we consider these resilencings as irreversible. The second key point is the *greedy heuristic* in the choice of the reactions to be silenced: *the higher the induced recovery of the growth rate, the higher the probability for the cell to adopt that resilencing.* We calculate these probabilities with the following equations.

For simplicity let us consider the case in which, while performing step 1 (from $s = 0$ to $s = 1$) of the adaptation dynamics, the cell has only two possible strategies (i.e. two possible resilencings) here called A and B (see Fig. S3A and B). For each of them, MOMA is applied with respect to $\mathbf{v}^{(0)}$ leading to $\mathbf{v}^{(\text{A})}$ and $\mathbf{v}^{(\text{B})}$ with the corresponding growth rates g_{A} and g_{B}. Without loss of generality we can assume $g_{\text{A}} > g_{\text{B}}$. According to our description of a *greedy regulation machinery*, strategy A is adopted by the cell with a higher probability than that of strategy B. We denote r_A and $r_B = 1 - r_A$ the two corresponding probabilities. This means that $r_A > r_B$ and that from an initial population of $Q(0)$ cells we have $r_A Q(0)$ cells with growth rate g_{A} and $r_B Q(0)$ cells with growth rate g_{B}. Considering

the elementary Population Dynamics equation $dQ(t)/dt = gQ(t)$, after a time Δt (the time interval between two consecutive steps) the two populations are:

$$\begin{aligned} Q_A(\Delta t) &= r_A Q(0) e^{g_\mathrm{A}\Delta t} \\ Q_B(\Delta t) &= r_B Q(0) e^{g_\mathrm{B}\Delta t}. \end{aligned} \tag{S4}$$

i.e. also the proliferation difference introduces a higher probability of finding cells that have adopted resilencing A (Fig. S3B). This is due to the exponential expression that, by the factor $\Delta t > 0$, converts the difference in terms of growth rates into a difference in terms of population of cells (i.e. $g_\mathrm{A} > g_\mathrm{B} \Rightarrow Q(0)e^{g_\mathrm{A}\Delta t} > Q(0)e^{g_\mathrm{B}\Delta t}$). For simplicity we decided to model the greedy heuristic of the regulatory machine (i.e. $g_\mathrm{A} > g_\mathrm{B} \Rightarrow r_A > r_B$) through the same exponential expression. Clearly, Δt is here replaced by an arbitrary parameter, denoted by β_reg. The resulting expressions for r_A and r_B are then:

$$\begin{aligned} r_A &= \frac{e^{g_\mathrm{A}\beta_\mathrm{reg}}}{e^{g_\mathrm{A}\beta_\mathrm{reg}} + e^{g_\mathrm{B}\beta_\mathrm{reg}}}; \\ r_B &= \frac{e^{g_\mathrm{B}\beta_\mathrm{reg}}}{e^{g_\mathrm{A}\beta_\mathrm{reg}} + e^{g_\mathrm{B}\beta_\mathrm{reg}}}. \end{aligned} \tag{S5}$$

Combining (S4) and (S5), the probabilities of finding a cell with strategy A or strategy B are given by

$$\begin{aligned} \mathbb{P}[A] &= \frac{Q_\mathrm{A}(\Delta t)}{Q_\mathrm{A}(\Delta t) + Q_\mathrm{B}(\Delta t)} = \frac{e^{g_\mathrm{A}(\beta_\mathrm{reg}+\Delta t)}}{e^{g_\mathrm{A}(\beta_\mathrm{reg}+\Delta t)} + e^{g_\mathrm{B}(\beta_\mathrm{reg}+\Delta t)}}; \\ \mathbb{P}[B] &= \frac{Q_\mathrm{B}(\Delta t)}{Q_\mathrm{A}(\Delta t) + Q_\mathrm{B}(\Delta t)} = \frac{e^{g_\mathrm{B}(\beta_\mathrm{reg}+\Delta t)}}{e^{g_\mathrm{A}(\beta_\mathrm{reg}+\Delta t)} + e^{g_\mathrm{B}(\beta_\mathrm{reg}+\Delta t)}}. \end{aligned} \tag{S6}$$

The two parameters are then combined by a simple summation that leads to a *single parameter* β for the entire model, namely

$$\beta = \beta_\mathrm{reg} + \Delta t. \tag{S7}$$

We would like to notice that, as long as the equation reproduces the greedy criterion $g_\mathrm{A} > g_\mathrm{B} \Rightarrow r_A > r_B$, other mathematical expressions can be used. We verified this by comparing the results from the exponential equation with the β parameter and the results from a probability calculated with the m-th order moment. Fig. 2a shows indeed a good similarity between the two results.

Parallelism with Statistical Physics. Derived Eq. (S6) has the same form as the Boltzmann distribution used in Statistical Physics. Indeed, defining the Hamiltonian as $H = -g$ and the β factor as $\beta = \beta_\mathrm{reg} + \Delta t$, the probability of being in state ω becomes $\mathbb{P}[\omega] = e^{-\beta H(\omega)}/Z$, where Z is the partition function of the canonical ensemble defined as $Z = \sum_{\omega\in\Omega} e^{-\beta H(\omega)}$ and Ω is the ensemble of all possible states of the systems [9]. Therefore, like in a typical minimization energy problem in Statistical Physics, we can think to the microorganism as an algorithm that is trying to minimize (maximize) the Hamiltonian H (the growth rate g); see also Fig. S12H-J. From this analogy we can also derive the information about the meaning of β: its value is related to the "temperature", i.e. the randomness of the dynamics. We refer to this interpretation for the choice of the value of β (see below).

The end-point $[\mathbf{v}^{(\mathbf{end})}, \mathbf{L}^{(\mathbf{end})}, \mathbf{U}^{(\mathbf{end})}]$. Equation (S6) can be applied to any number of possible strategies and to any step s of the trajectory. Thus, through an iterative use of resilencing, the growth is gradually recovered. Please note that, since there exist resilencings which cause negative effect on cell proliferation, a non-monotonic increasing trajectory of the growth rate is always possible.

We define then the end-point of the adaptation trajectory the state for which *none* of the possible resilencings can provide any further increase of the growth rate, i.e. there is no advantage for the cell in resilencing other reactions.

Markov chain. The adaptation dynamics we have described form a Markov Chain (see Fig. S3C) for which the probabilities at all possible steps are well defined and computable, and whose states are represented by the triplet

$$[\mathbf{v}^{(s)}, \mathbf{L}^{(s)}, \mathbf{U}^{(s)}] \in \mathbb{R}^{3r}.$$

It is worth noting that the sequence of vectors $\{\mathbf{v}^{(s)}\}_{s=0,1,2...}$ without $\mathbf{L}^{(s)}$ and $\mathbf{U}^{(s)}$ is not sufficient to describe the dynamics. Indeed, similarly to other dynamical approaches [12, 1], by expanding the space from $\mathbb{R}^r$ to $\mathbb{R}^{3r}$ we make the process Markovian ($\{\mathbf{v}^{(s)}\}_{s=0,1,2...}$ alone is not Markovian).

At each step, the transition probabilities must be calculated as the system evolves. Because of the high number of possible trajectories (for the cases analysed in this work we estimate that more than 10^{30} trajectories are possible) an exhaustive sampling is not practically feasible. Therefore the space is sampled using a kinetic MonteCarlo method [3]. At a generic step s a random number is extracted according to the distribution of the transition probabilities, and the corresponding resilencing is introduced. The system passes then from state $[\mathbf{v}^{(s)}, \mathbf{L}^{(s)}, \mathbf{U}^{(s)}]$ to state $[\mathbf{v}^{(s+1)}, \mathbf{L}^{(s+1)}, \mathbf{U}^{(s+1)}]$ and the procedure is iterated until an end-point is reached (see Fig. S3C). The resulting trajectory consists of a sequence of accepted enzyme resilencings ($\mathbf{L}^{(s)}$ and $\mathbf{U}^{(s)}$) together with the corresponding metabolic fluxes ($\mathbf{v}^{(s)}$). The time-scale of the dynamics in Fig. 1 of the main text is obtained by t [days] $= 1.2s$ [step number of the recursive procedure] (i.e. $\Delta t \approx 30$ h).

Many random realizations of the dynamics can be generated. All the calculations in the paper are performed computing N =500 trajectories for each condition. Averaging over these N trajectories we obtain the mean dynamics of the growth rate $g(s)$ and the corresponding standard deviations.

Normalization of the reaction fluxes. For a comparison with the ^{13}C-based measurements, we normalize the fluxes in a way to preserve also the important ratio between the values in the wild-type and the values in the mutant. In the following, $\mathbf{v}^{\text{exp}}$ and $\mathbf{v}^{\text{comp}}$ denote the experimental and the computed (not normalized) vectors of fluxes for the mutant strain, whereas $\mathbf{w}^{\text{exp}}$ and $\mathbf{w}^{\text{comp}}$ denote the same for the wild-type (see Fig. S6 for the results on the wild-type strain). The corresponding normalized fluxes $\mathbf{v}^{\text{norm}}$ and $\mathbf{w}^{\text{norm}}$ are calculated as follows:

$$\mathbf{v}^{\text{norm}} = \mathbf{v}^{\text{comp}} \left(\frac{\mathbf{v}^{\text{exp}} + \mathbf{w}^{\text{exp}}}{\mathbf{v}^{\text{ comp}} + \mathbf{w}^{\text{comp}}} \right);$$

$$\mathbf{w}^{\text{norm}} = \mathbf{w}^{\text{comp}} \left(\frac{\mathbf{v}^{\text{exp}} + \mathbf{w}^{\text{exp}}}{\mathbf{v}^{\text{ comp}} + \mathbf{w}^{\text{comp}}} \right).$$

The formulas have been applied to the results obtained by GRAM, MOMA, ROOM and pFBA.

1.2 Choice of the parameter β

As mentioned earlier, from Statistical Physics we know that the value of β is related to the "temperature" of the system, i.e. to the randomness of the dynamics. In particular, low values of β imply high randomness, and high values of β tend to give deterministic dynamics. Since we would like to include alternative trajectories without generating results with too high variability, we looked for a reasonable compromise for the value of this parameter. For this purpose we have explored how the variance of the growth rate at the end-point of the trajectories is affected by β. For a fixed value of β, we simulated the set C of all (independent) experimental conditions considered in this work (knockouts plus carbon sources) and then we computed the averaged relative variance, $\bar{\sigma}^2_{\mathrm{rel}}$ as the sum of the relative variance of each of these (independent) conditions:

$$\bar{\sigma}^2_{\mathrm{rel}}(\beta) = \sum_{i \in C} \frac{\sigma_i^2(\beta)}{[\bar{g}_i^{(\mathrm{end})}(\beta)]^2} \tag{S8}$$

where for the i-th experiment we used the common variance estimator

$$\sigma_i^2(\beta) = \frac{1}{N-1} \sum_{j=1}^{N} \left[g_i^{(\mathrm{end})}[j,\beta] - \bar{g}_i^{(\mathrm{end})}(\beta) \right]^2 \tag{S9}$$

and $\bar{g}_i^{(\mathrm{end})}(\beta)$ is the mean value over the $N = 500$ simulated trajectories of experiment i with that β. Figure S4 reports the results of this analysis: as expected, increasing the randomness (i.e. reducing β) increases the variance monotonically. However, two different regimes are present: a "flat-low regime" showing only small changes to the already low values of variance, and a "steep-high regime" with significant changes and high values of variance. In order to have a reasonably limited variability of the outcomes while still being far from deterministic trajectories, we decided to choose $\beta = 200$ h, i.e. a value in the flat-low regime but close to the transition between the two regimes. As an example, an evaluation of the effect of β on the non-trivial GLU-*tpi* condition is reported in Fig. 2a in the main text.

We would like to stress the fact that, as clearly indicated by Eqs. (S8)-(S9), the choice of the value of β has not been dictated by an optimal fit of the results with respect to experimental data but by a simple consideration on the amount of randomness. Therefore, all the results of our simulations should be viewed as real model predictions and the comparison with the experimental data as a stringent test for our principle.

2 SUPPORTING RESULTS

2.1 Tests about the importance of greediness.

We verified the role of greediness with the following tests.

- **Alternative expression for the probability.** In order to verify that the results are not an artefact due to the usage of the Boltzmann equation (S6), we tested whether the quality of the outcomes is preserved by the use of other equations. As example of alternative estimation of the probability, we considered the use of the m-th moment:

  $$\mathbb{P}[\omega] = \frac{(g_\omega)^m}{\sum_{\omega\in\Omega}(g_\omega)^m}. \tag{S10}$$

  We would like to note that both expressions reproduce the greedy heuristic $g_A > g_B \Rightarrow \mathbb{P}[A] > \mathbb{P}[B]$. Clearly, this is the only requirement the equation must fulfil. We choose GLU-*tpi* as testing case because of its non-trivial adjustment (i.e. a sub-optimal recovery of the growth rate). The comparison of the results is reported in Fig. 2a in the maintext. The overlap between the two sets of results supports the absence of artefact in the use of the Boltzmann equation and the generality of the greedy criterion.

  It is also interesting to notice that the value of $\beta = 200$ h corresponds approximately to $m = 2$ (see Fig. 2a in the maintext). Therefore, Eq. (S10) has the same expression as a Hill equation with cooperative index equal to 2 (a value widely used in the literature). The equivalence between the two expressions has been derived with more details in the maintext in "An example of a greedy regulatory motif" at the end of the Results section.

- **Comparison with deterministic and random models.** Since our trajectories are about 18-24 steps long, the temporal scale of 40-50 days of adaptation indicates that each iterative step corresponds to a Δt of about 30 hours and then, from Eq. (S7), $\beta_{\text{reg}} = \beta - \Delta t = 170\ \text{h}^{-1}$. This already suggests that the role of greediness in the dynamics is more important that the effect of a better "fitness" (the induced proliferation advantage over the time).
  Moreover, by the analogy between our probability estimation and the Boltzmann distribution, the factor β_{reg} can be related to the amount of randomness in our Markov Chain. We then tested the case when $\beta_{\text{reg}} \to \infty$ (a deterministic greedy choice of the resilencing, i.e. the resilencing which gives the highest growth rate is always adopted) and when $\beta_{\text{reg}} = 0\ \text{h}^{-1}$(a completely random choice in which all resilencings become equiprobable, i.e. the greedy criterion is removed). A comparison of the two cases with respect to the standard method ($\beta_{\text{reg}} = 170\ \text{h}^{-1}$) is reported in Fig. 2b-d in the main text and show that the deterministic model is still able to provide good results (though naturally, it does not reproduce the appearance of two phenotypes in GLU-*pgi*) whereas the removal of the greediness causes a complete loss of the prediction power.

- **Comparison with shuffled and merged sequence of resilencings.** To prove the importance of greediness in choosing the temporal order, we recomputed the end-point of the trajectories obtained with the standard method assuming that the sequences of resilencing are randomly shuffled or merged in a single step. In both cases the new results are no longer in agreement with the experimental data (see Fig. 2e-f in the main text).

- **Comparison with available methods.** We compared also our end-points (final growth rate) with the results derived from MOMA, ROOM and pFBA. Plots in Fig. S5 clearly indicate the better performance of our method. An additional confirmation is obtained also on the basis of the ^{13}C-based reaction fluxes as reported in Table S2. Although promising, we have not considered the Pareto optimality proposed in [14] since it provides a range of values for the fluxes and not a specific point in the FBA polytope.

2.2 Mutations

The use of $r_A = r_B$ ($\beta_{\text{reg}} = 0$, i.e. no greediness) would be ideal to describe the appearance of (random) mutations that stop a reaction. However, mutations are rare in the considered time scale, especially those which cause a stop of a reaction: most of the resilencings are indeed induced by the regulatory machine of the cell [5, 2]. Similar consideration applies to mutations that trigger an increase of the enzymatic activity. Nevertheless, this type of mutation can still be described by GRAM. Indeed, since the only limiting factor in the network is the carbon source uptake, reaction fluxes are never limited by the lower/upper bounds and their absolute value can always increase during MOMA projection. We report here two examples.

The ≈2.5-fold higher enzymatic efficiency [5] measured in the *in vivo* activity assay on the enzymes in the methylglyoxal pathway in the case of Δ*tpi* is indeed reproduced in our simulations by fluxes 3.6 times higher in the mutant than in the wild-type.

Experimental evidence on Δ*pgi* shows that the activation of the pentose phosphate pathway and of isocitrate dehydrogenase causes an overproduction of NADPH. This is compensated by a mutation that stops membrane-bound transhydrogenase and activates soluble NADPH transhydrogenase [5, 7, 2]. In our trajectories for the wild-type, the production and consumption of NADPH by the different metabolic pathways is almost balanced: the consumption exceeds the production by less than 1 mmol/gh. This small difference is compensated by the synthesis of NADPH by the membrane-bound transhydrogenase (NADH → NADPH). In the case of the *pgi* knockout, instead, both phenotypes show an excess in the production of NADPH due to the pentose phosphate pathway and isocitrate dehydrogenase activation. To recover a correct balance of NADPH, the membrane-bound transhydrogenase is silenced and the soluble NADPH transhydrogenase activated (NADPH → NADH), especially for the low growth phenotype. The ratio of the predicted flux between the two phenotypes is 2.5 and the experimental ratio is 2.7. As described in the main text, an additional confirmation is provided by the effect of the timing of this resilencing (see Fig. 4d).

2.3 An example of network with suboptimal end-point

As mentioned in the main text, because of its simplicity, in the minimal model of Fig. 5 local information is equal to global information, i.e. the system never shows a suboptimal end-point. However, the simple equations where useful to identify the constraint on the Hill constants that is necessary for a greedy regulation, in particular if $\nu_A > \nu_B$ then $H_A > H_B$ should hold. In order to have a general confirmation of this finding and to support the idea of the implementation of greediness by negative feedbacks, we built a more complex network where local and global information are different. The construction of the network has been inspired by the bifurcation study in the GLU-*tpi* dynamics. A sketch of this network is reported in Fig. S10A.

The system contains the same implementation of greediness by negative feedbacks and corresponding Hill equations we used in the toy model of Fig. 5. The two initial alternative resilencings (i.e. the bifurcation between reaction A or B) have been inherited from that minimal model. They now synthesize the intermediate compounds I_1 and I_2 with stoichiometric efficacy ν_A and ν_B, respectively, such that $\nu_A > \nu_B$. Downstream of each reaction, two new regulated processes have been added to transform the intermediate compounds into the metabolites M_1 and M_2 but with an opposite stoichiometry, i.e. the pathway downstream of reaction A is less efficient that the pathway downstream of reaction B. This is obtained by requiring that $\nu_A \max\{\nu_C, \nu_D\} < \nu_B \max\{\nu_E, \nu_F\}$. Because of the concatenated processes, the first regulation on A *vs* B does not know *a priori* the possible effect of the downstream regulations thus, for choosing whether to silence reaction A or reaction B, the cell can use only local information. Because of this myopic choice, local information differs from global information and the system can evolve to a suboptimal solution. It is interesting to note the similarity between this dynamics and the bifurcation in GLU-*tpi* where reaction A = *transketolase II*, reaction B = *phosphoglucose isomerase*, with glycolysis and pentose phosphate pathway as the two downstream processes, respectively.
The differential equations for this network are the following:

$$\begin{cases} \frac{d[\mathrm{P}]}{dt} &= u(t) - k_A \frac{H_A^2}{H_A^2+[\mathrm{M}]^2}[\mathrm{P}] - k_B \frac{H_B^2}{H_B^2+[\mathrm{M}]^2}[\mathrm{P}]; \\ \frac{d[\mathrm{M}]}{dt} &= k_A \frac{H_A^2}{H_A^2+[\mathrm{M}]^2}[\mathrm{P}] + k_B \frac{H_B^2}{H_B^2+[\mathrm{M}]^2}[\mathrm{P}] - \delta[\mathrm{M}]; \\ \frac{d[\mathrm{I}_1]}{dt} &= \nu_A k_A \frac{H_A^2}{H_A^2+[\mathrm{M}]^2}[\mathrm{P}] - k_C \frac{H_C^2}{H_C^2+[\mathrm{M}_1]^2}[\mathrm{I}_1] - k_D \frac{H_D^2}{H_D^2+[\mathrm{M}_1]^2}[\mathrm{I}_1]; \\ \frac{d[\mathrm{M}_1]}{dt} &= \nu_C k_C \frac{H_C^2}{H_C^2+[\mathrm{M}_1]^2}[\mathrm{I}_1] + \nu_D k_D \frac{H_D^2}{H_D^2+[\mathrm{M}_1]^2}[\mathrm{I}_1] - h[\mathrm{M}_1]; \\ \frac{d[\mathrm{I}_2]}{dt} &= \nu_B k_B \frac{H_B^2}{H_B^2+[\mathrm{M}]^2}[\mathrm{P}] - k_E \frac{H_E^2}{H_E^2+[\mathrm{M}_2]^2}[\mathrm{I}_2] - k_F \frac{H_F^2}{H_F^2+[\mathrm{M}_2]^2}[\mathrm{I}_2]; \\ \frac{d[\mathrm{M}_2]}{dt} &= \nu_E k_E \frac{H_E^2}{H_E^2+[\mathrm{M}_2]^2}[\mathrm{I}_2] + \nu_F k_F \frac{H_F^2}{H_F^2+[\mathrm{M}_2]^2}[\mathrm{I}_2] - h[\mathrm{M}_2]. \end{cases} \tag{S11}$$

We ran numerical simulations using the parameters reported in Fig. S10A and studied the effect of the two Hill constants H_A and H_B. The single silencing of reaction A (or B) gives $g_A = 0.62$ (or $g_B = 1.12$). Therefore, from the results from toy model in the main text, we know that if $H_A > H_B$ then the regulation of A *vs* B is greedy, i.e. B is silenced with higher probability. Results with $H_A = 9$ and $H_B = 1$ are plotted in Fig. S10C and show that, also in the case of a more complex network with a suboptimal end-point, negative feedbacks represent a possible biological implementation of the greedy strategy (reaction B is silenced whereas reaction A is kept active). Moreover, because of the myopic chioce, the system ends at the suboptimal end-point, as expected. We would like also to note that, as shown in Fig. S10B-C, in order to make the system reach the optimal state, a non-greedy choice ($H_A < H_B$) must be performed (like the silencing of phosphoglucose isomerase in GLU-*tpi*).

2.4 Clustering of the dynamics.

The distance $d(s)$: Considering the analogy between our systems and the energy minimization problem, we interpret the regulatory dynamics as a trajectory of a particle in the polytope. Let $\mathbf{v}^{(0)}$ and $\mathbf{v}^{\text{opt}}$ be the initial point and the pFBA optimal point for a given experimental condition c. At any step s of a trajectory j, we calculate the distance between $\mathbf{v}^{(\text{s})}[j]$ and the line segment that links $\mathbf{v}^{(0)}$ and $\mathbf{v}^{\text{opt}}$, called "shortest path" in the following (see "distance" reported in Fig. S12H). This is obtained by the following expression

$$d_c^{(\text{s})}[j] = ||\mathbf{v}^{(\text{s})}[j] - [(1-\alpha(s,j))\mathbf{v}^{(0)} + \alpha(s,j)\mathbf{v}^{\text{opt}}]||_2, \tag{S12}$$

where

$$\alpha(s,j) = \frac{(\mathbf{v}^{(\text{s})}[j] - \mathbf{v}^{(0)})^T(\mathbf{v}^{\text{opt}} - \mathbf{v}^{(0)})}{||\mathbf{v}^{\text{opt}} - \mathbf{v}^{(0)}||_2^2}.$$

An average over $j = 1, \ldots, N$ provides the dynamics $d_c(s)$.

Plots of $d(t)$ are reported in Fig. S11B. By definition, $d(s)$ indicates how closely the metabolic adjustment follows the "shortest path". It follows that $d(t)$ starts from zero and if the systems arrives to the optimal point (or close to it), $d(t)$ goes back to zero (or close to it). Therefore, unless the dynamics follow the "shortest path", $d(t)$ increases during the first part of the trajectory and decreases in the second part. In case a suboptimal point is reached, $d(t)$ stays high, as for GLU-*ppc*, GLU-*tpi* and GLU-*pgi*.

Although not fully intuitive, this distance has been used because it provides some additional information about the regulatory dynamics. For instance, the fact that $d(t)$ deviates significantly from the shortest path suggests that, instead of moving straight towards the optimal point, cells need to do some preliminary adjustments to the metabolism. This is the case, for example, of the resilencing of NADPH transhydrogenase analysed in GLU-*pgi*. As described in the last paragraph in "Explanations from the greedy hypothesis" of the main text (and Fig. 4d), this important resilencing is never done at the beginning of the trajectory, i.e. it must wait for other preliminary regulations to first be adopted by the cell.

It is worth noting that the increase of $d(t)$ in the first part of the trajectory does not necessarily imply that the system is moving farther from the optimal point. To show this, the more intuitive distance $f(t)$ has been calculated: in particular $f(t)$ is the distance between the trajectory point at time t and the optimal point, so it measures how far the systems is from the optimal point:

$$f(t) := ||\mathbf{v}^{\text{opt}} - \mathbf{v}^{(\text{t})}||_2.$$

Results show a monotonic decrease of $f(t)$, which means that the system constantly approaches the optimal point: often it is able to reach it (or to arrive very close to it) but sometimes it is trapped in a local minimum (see Fig. S11C).

Clustering From the ensemble of all the considered experimental conditions c, we construct a distance tree. The corresponding matrix M of distances between each pair (c_i, c_j) of experimental conditions is calculated as follows:

$$M_{i,j} = ||d_{c_i} - d_{c_j}||_2 = \sqrt{\sum_s \left[d_{c_i}(s) - d_{c_j}(s)\right]^2}.$$

The result reported in Fig. S11A shows that there are three possible classes of trajectories:

I. $d(t)$ remains low throughout the trajectory: the adjustment of the metabolism moves directly to the maximal biomass production following closely the shortest trajectory (i.e. the line connecting $\mathbf{v}^{(0)}$ to $\mathbf{v}^{\text{opt}}$);

II. $d(t)$ increases and then decreases to a low value: the adjustment initially deviates from the straight direction but at the end it reaches the pFBA optimum $\mathbf{v}^{\text{opt}}$;

III. $d(t)$ increases and never returns near zero: the adjustment deviates and is no longer able to reach $\mathbf{v}^{\text{opt}}$.

Class II is the most populated: among all the conditions we analysed, 3 belong to class I, 12 to class II and 3 to class III. It is worth noting that for all the cases belonging to class III, the recovery of the growth rate is only partial. An example of each of the three classes is reported in Fig. S12 (namely, *frd* knockout on lactate, *pck* on glucose and *tpi* on glucose). The last row of panels of Fig. S12 shows the analogy with the energy minimization problem (Hamiltonian $H = -g$). Viewed as trajectories of a particle in the polytope over a energy landscape, classes I and II might have a single valley (at least on the part explored from our $\mathbf{v}^{(0)}$) whereas class III might have a second valley with a local minimum of energy, where most of the trajectories remain trapped.

References

[1] Giovanni Bussi, Alessandro Laio, and Michele Parrinello. Equilibrium free energies from nonequilibrium metadynamics. *Physical review letters*, 96(9):090601, 2006.

[2] P. Charusanti, T.M. Conrad, E.M. Knight, K. Venkataraman, Fong. N.L., Y. Xie, B. amd Gao, and B.Ø. Plasson. Genetic basis of growth adaptation of Escherichia coli after deletion of pgi, a major metabolic gene. *PLoS Genetics*, 6(11):e1001186, 2010.

[3] David Roxbee Cox and Hilton David Miller. *The Theory of Stochastic Processes.* Wiley, New York, 1965.

[4] S.S. Fong, J.Y. Marciniak, and B.Ø. Palsson. Description and interpretation of adaptive evolution of Escherichia coli K-12 MG1566 by using a genome-scale in silico metabolic model. *Journal of Bacteriology*, 185(21):6400–6408, 2003.

[5] S.S. Fong, A. Nanchen, B.Ø Plasson, and U. Sauer. Latent pathway activation and increased pathway capacity enable Escherichia coli adaptation to loss of key metabolic enzymes. *Journal of Biological Chemistry*, 281(12):8024–8033, 2006.

[6] Stephen S Fong and Bernhard Ø Palsson. Metabolic gene–deletion strains of Escherichia coli evolve to computationally predicted growth phenotypes. *Nature genetics*, 36(10):1056–1058, 2004.

[7] Q. Hua, C. Yang, T. Baba, H. Mori, and K. Shimizu. Response of central metabolism in Escherichia coli to phosphoglucose isomersare and glucose-6-phosphate dehydrogenase knockouts. *Journal of Bacteriology*, 185(24):7053–7067, 2003.

[8] Qiang Hua, Andrew R Joyce, Bernhard Ø Palsson, and Stephen S Fong. Metabolic characterization of Escherichia coli strains adapted to growth on lactate. *Applied and environmental microbiology*, 73(14):4639–4647, 2007.

[9] Kerson Huang. *Introduction to Statistical Physics.* CRC Press, New York, 2001.

[10] Joonhoon Kim and Jennifer L. Reed. RELATCH: relative optimality in metabolic networks explains robust metabolic and regulatory responses to perturbations. *Genome biology*, 13(9):R78, Jul 2012.

[11] Joonhoon Kim and Jennifer L. Reed. Refining metabolic models and accounting for regulatory effects. *Current opinion in biotechnology*, 29:34–38, Oct 2014.

[12] Alessandro Laio and Francesco L Gervasio. Metadynamics: a method to simulate rare events and reconstruct the free energy in biophysics, chemistry and material science. *Reports on Progress in Physics*, 71(12):126601, 2008.

[13] A.E. Motter, N. Gulbahce, E. Almaas, and A.L. Barabási. Predicting synthetic rescues in metabolic networks. *Molecular Systems Biology*, 4(1), 2008.

[14] Robert Schuetz, Nicola Zamboni, Mattia Zampieri, Matthias Heinemann, and Uwe Sauer. Multidimensional optimality of microbial metabolism. *Science*, 336(6081):601–604, May 2012.

[15] D. Segré, D. Vitkup, and G.M. Church. Analysis of optimality in natural and perturbed metabolic networks. *Proceedings of the National Academy of Sciences USA*, 99 (23):15112–15117, 2002.

[16] T. Shlomi, O. Berkman, and E. Ruppin. Regulatory on/off minimization of metabolic flux changes after genetic perturbations. *Proceedings of the National Academy of Sciences USA*, 102 (21):7695–7700, 2005.

Figure S1: **Optimal and suboptimal recovery of the growth rate.** The analysis of all the different perturbations (knockout and carbon-source) considered in this work shows that in about 20% of the conditions the recovery of the growth rate is significantly sub-optimal.

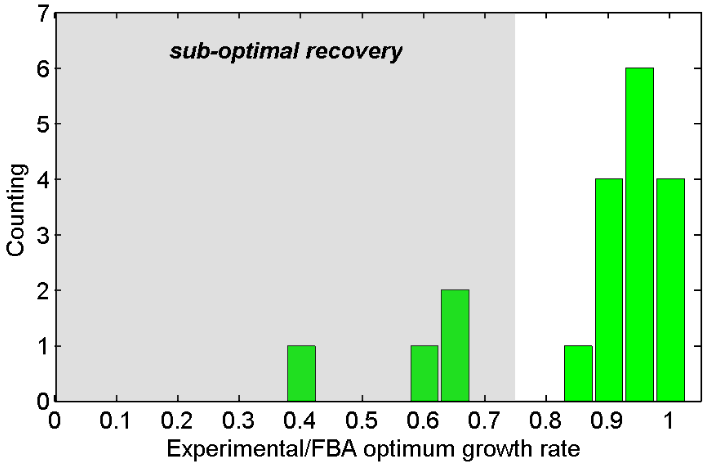

Figure S2: **Stoichiometric network. (A)**: Simplified sketch of the *E. coli* core metabolic network with the most important pathways. Red boxes indicate the knockouts considered in this work. DHAP: dihydroxyacetone phosphate; GAP: glyceraldeide 3-phosphate; PEP: phosphoenolpyruvate. **(B)**: Main features of the network. **(C)**: Rescaling of the upper-bounds for the exchange processes of carbon sources with respect to measured Substrates Uptake Rate (SUR, see Supplementary Table 1 of [6]) in order to reproduce the real growing conditions.

A

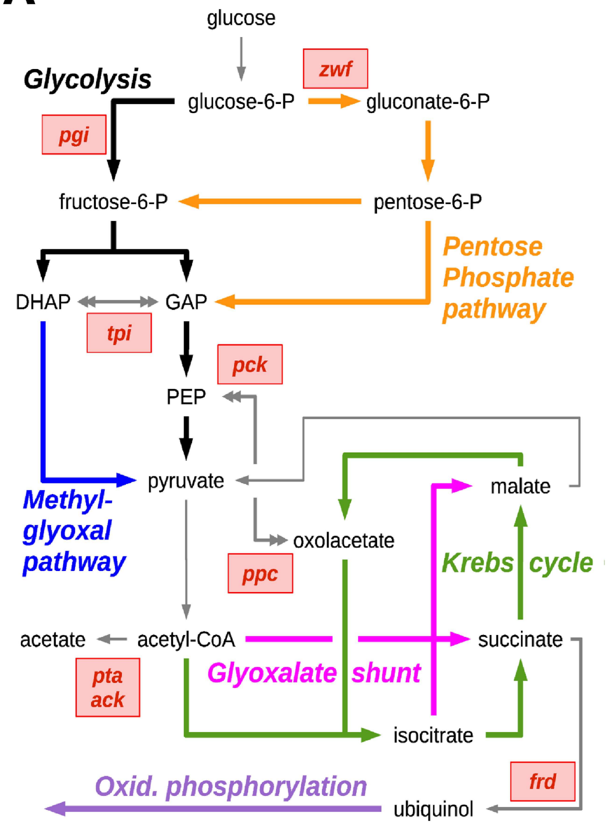

B

No. of reactions	96
No. of metabolites	72
No. of pathways	8
No. of compartments	2

C

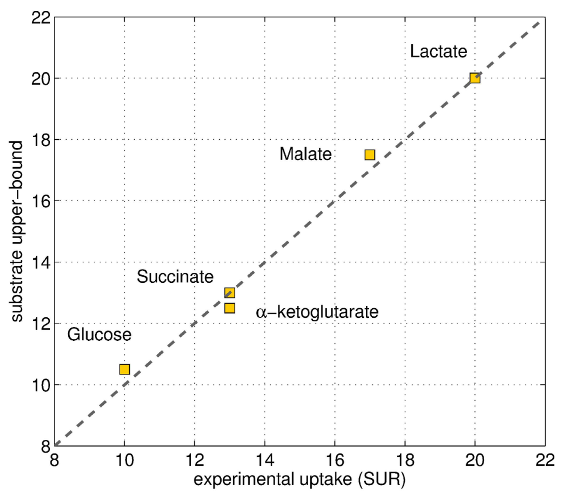

Figure S3: **Description of the method. (A)**: Sketch of the polytope reduction (from $W(\mathbf{L},\mathbf{U})$ to $W(\mathbf{L}^{(0)},\mathbf{U}^{(0)})$) and of the consequent MOMA L^2-projection (from blue $\mathbf{v}^{\text{pFBA}}$ to red dot $\mathbf{v}^{(0)}$) after the knockout. Two possible enzyme resilencings, A or B, at step 1 are also represented: green or magenta dotted lines respectively with the corresponding MOMA projections to $\mathbf{v}^{(A)}$ or $\mathbf{v}^{(B)}$. **(B)**: Definition of the probability of the resilencings A and B (same color code as in previous panel) as a combination of the greedy regulatory effect r_A *vs* r_B ($g_A > g_B \Rightarrow r_A > r_B$) and the basic population dynamics $dQ/dt = gQ$ over the time interval Δt. The probability of finding resilencing A at step 1 is equal to $\mathbb{P}[A] = Q_A(\Delta t)/[Q_A(\Delta t) + Q_B(\Delta t)]$. **(C)**: Markov chain scheme obtained from recursive resilencing (same color code as previous panels): at each step, MOMA is applied. Black arrows indicate a trajectory; grey arrows indicate alternative choices of the resilencing, i.e. alternative phenotypes. Thickness of the arrow indicates the probability of the choice.

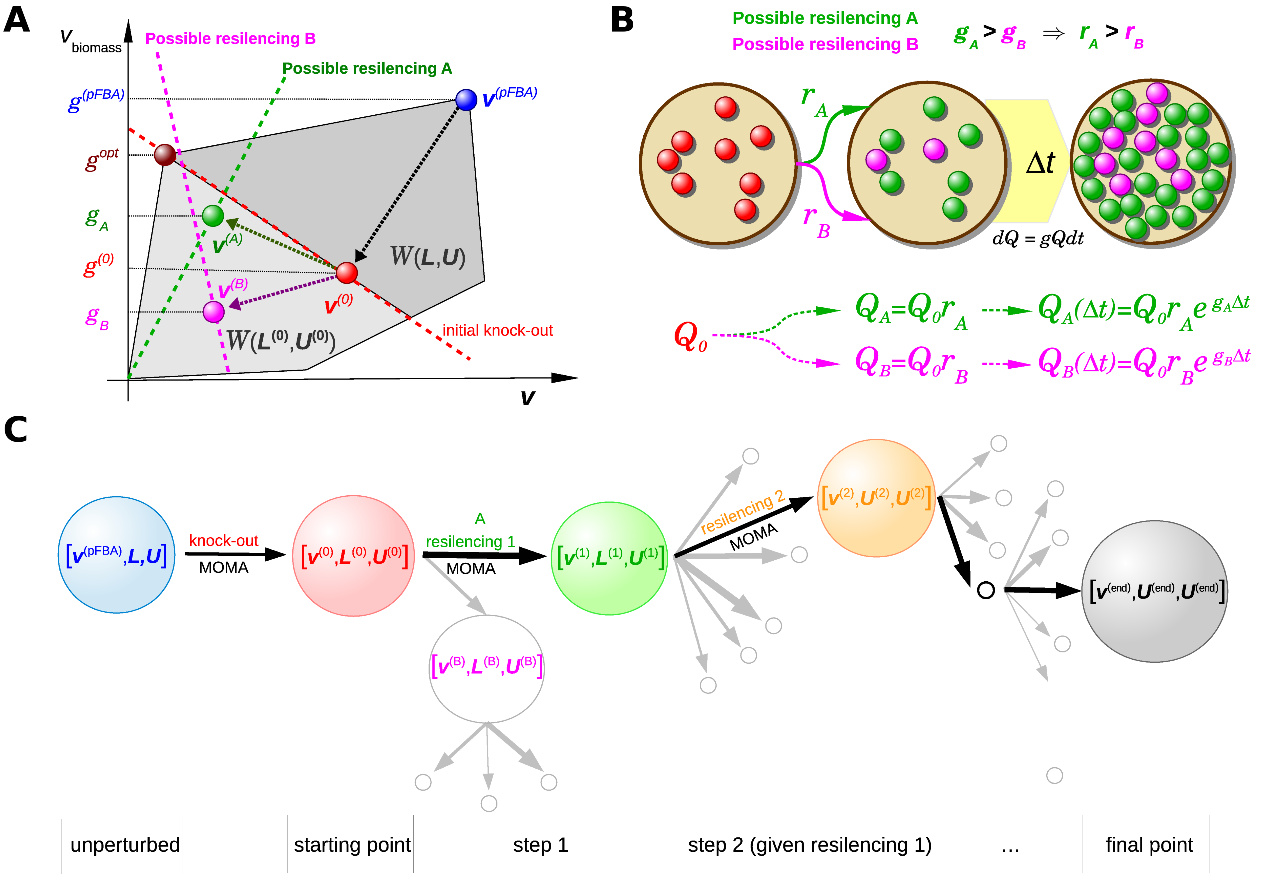

Figure S4: **Choice of value for the β factor.** Dots represent the results variability estimated by Eq. (S8) at various values of β. The blue dashed line indicates the "steep-high regime" whereas the red dashed line indicates the "flat-low regime". The thick arrow points to the value used in all our simulations, i.e. β=200 h. This value has been chosen in order to have a certain amount of randomness but still in the flat-low regime. The two small arrows point to the variance obtained by using the two single components of β alone, i.e. $\beta = \Delta t_{\text{real}}$=30 h (denoted by "time") and $\beta = \beta_{\text{reg}}$=170 h ("regulation").

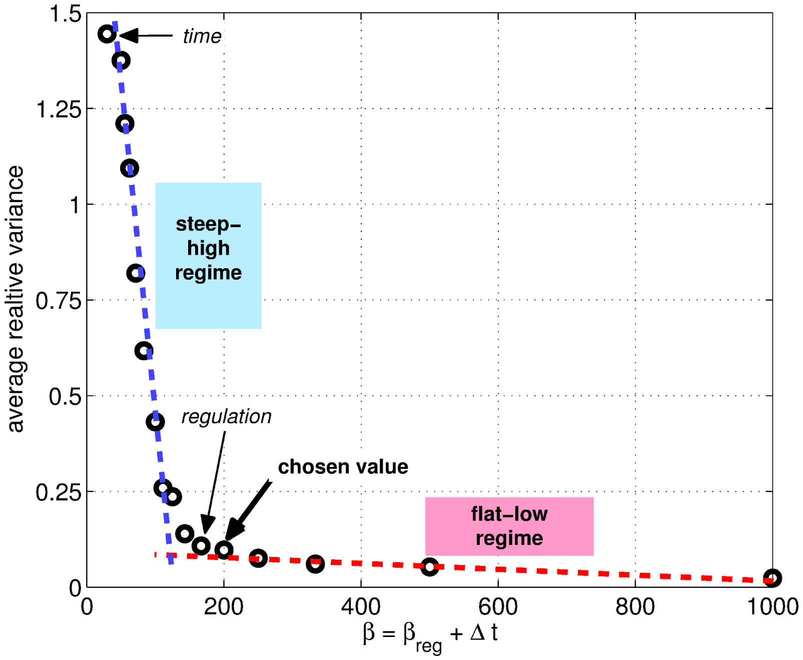

Figure S5: **Comparison with some methods from the literature.** Correlation (graphical and RMSD) between experimental growth rates from [6] and results obtained with various FBA methods confirms the best performance of GRAM. Color code for knockouts: red=*ack*, green=*frd*, violet=*zwf*, yellow=*ppc*, blue=*pck*, orange=*tpi*; legend for carbon sources: α-ketoglutarate (AKG, △), glucose (GLU, ○), lactate (LAC, □), malate (MAL, ◇) and succinate (SUC, ▽). Growth rates in h^{-1}.

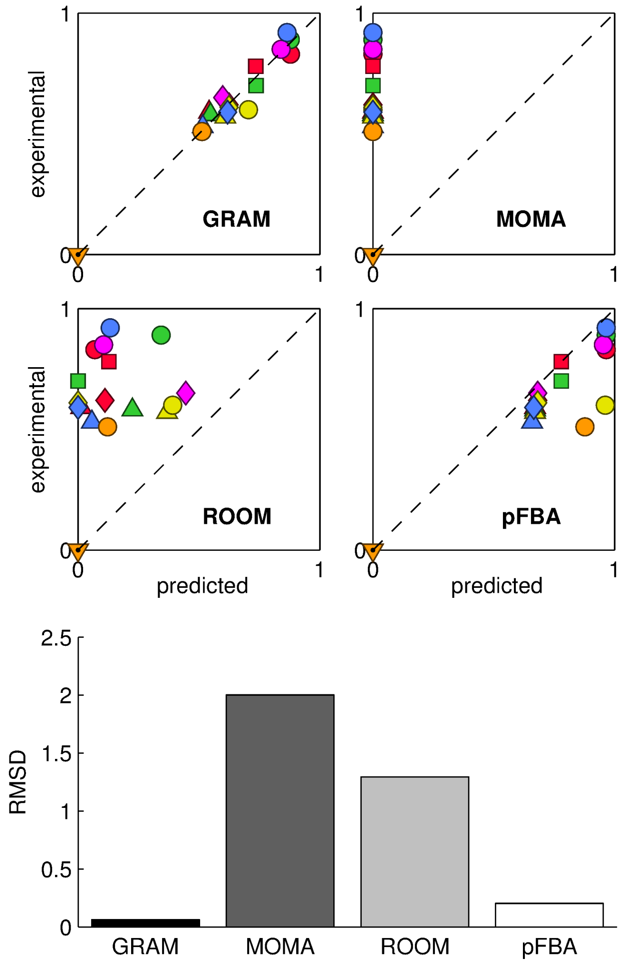

Figure S6: **Results for wild-type.** For comparison purposes, simulations have also been performed for the wild-type strain of *E.coli* for which the only perturbation is the switch from the Luria-Bertani rich medium to the single carbon source medium. Since the results (both theoretical and experimental) do not show any peculiar dynamics, we report here only the growth rate recovery and the agreement between predicted and measured reaction fluxes. **(A-E):** Dynamics in different carbon sources. **(F):** Validation by comparison with measurement of reaction fluxes in glucose (experimental data from [5]); RMSD=0.170.

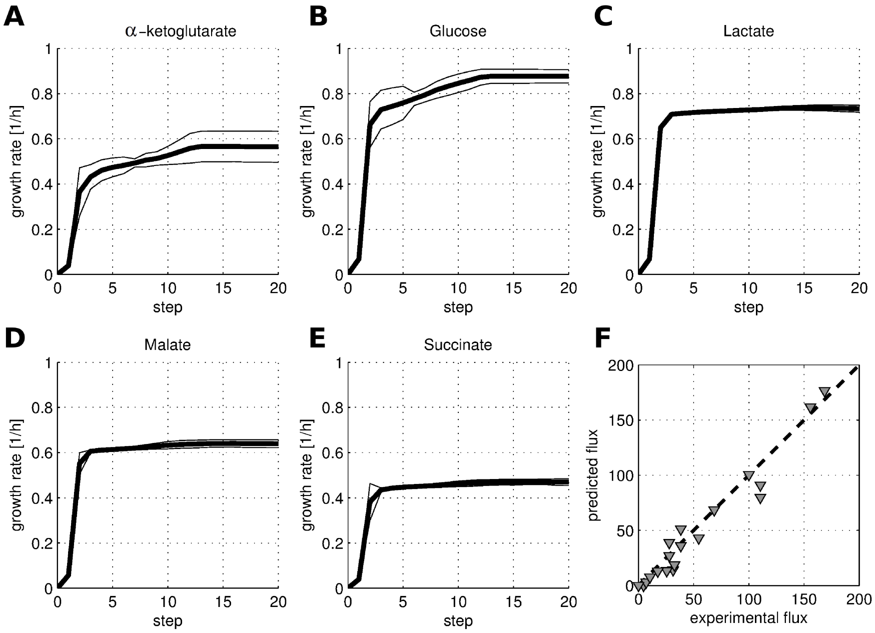

Figure S7: **Activated and silenced pathways for GLU-*pgi***. Coloured arrows and names indicate active processes. Coloured arrows and names indicate active processes. Red crosses indicate key silencings (**A**): Low growth rate phenotype which has resilenced glyoxalate shunt (**B**): High growth rate phenotype which has resilenced Krebs cycle and acetate secretion.

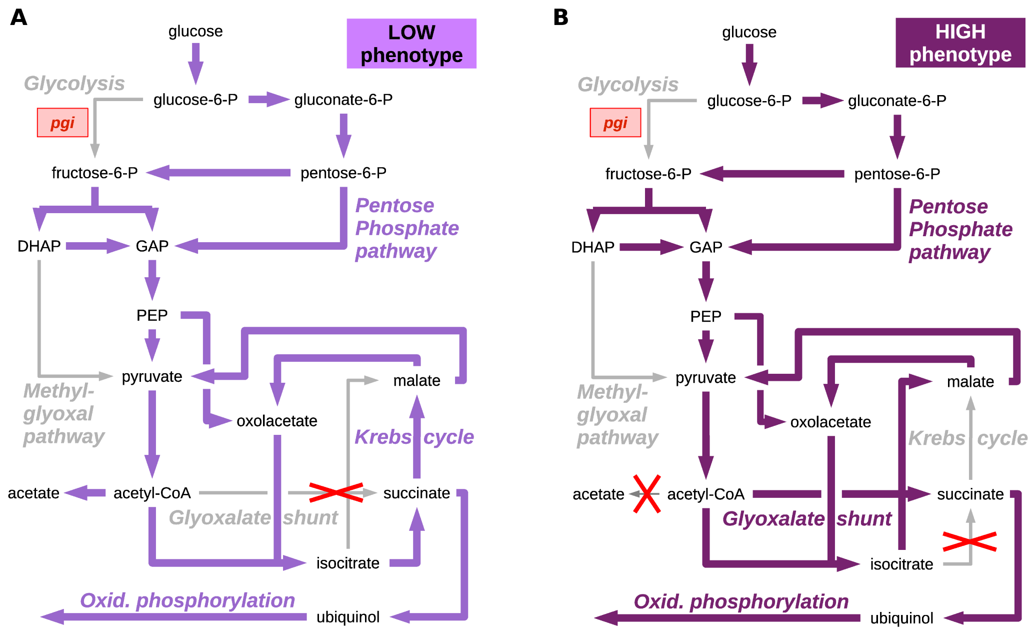

Figure S8: **Distribution of the standard deviation of the resilencing time for each reaction in each experimental condition.** Results obtained in the simulations from the ensemble of all the experimental conditions considered in the study. The distribution is bimodal: a first group is located at low values of variance (less than 2 days, approximately) and represents reactions that have a restricted and crucial resilencing time (see the example of membrane NADPH transhydrogenase in Fig. 4 of the maintext). A second broader peak centred at high value represents reactions for which the resilencing time is not crucial.

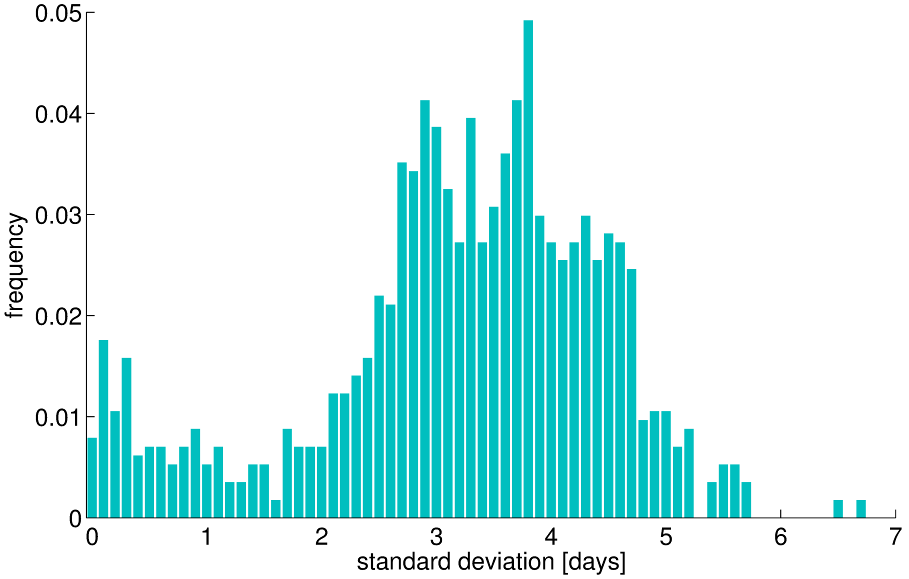

Figure S9: **An example of a greedy network motif.** Simulations from the model of Eq. (3) in the maintext. **(A):** Dynamics in case of resilencing of more efficient reaction A. The corresponding value of $g_A = 1.7$ is obtained. **(B):** Dynamics in case of resilencing of less efficient reaction B. The corresponding value of $g_B = 5.0$ is obtained. **(C):** final growth rate for different values of H_A and H_B. White crosses and numbers indicate the conditions used in panels D-F. **(D):** Dynamics with $H_A = 20$ and $H_B = 2$. **(E):** Dynamics with $H_A = g_B/h = 10$ and $H_B = g_A/h = 3.3$. **(F):** Dynamics with $H_A = H_B = 8$. **(G):** Effect of the cooperative index (i.e. moment order) on the final growth rate. Similar results are obtained with different moment order m in GRAM (see Fig. 2a in the maintext). This result reinforce the similarity between the two formalisms already suggested by Eq. (4) in the maintext. **(I):** Values of the model parameters for Eqs. (3) in the maintext.

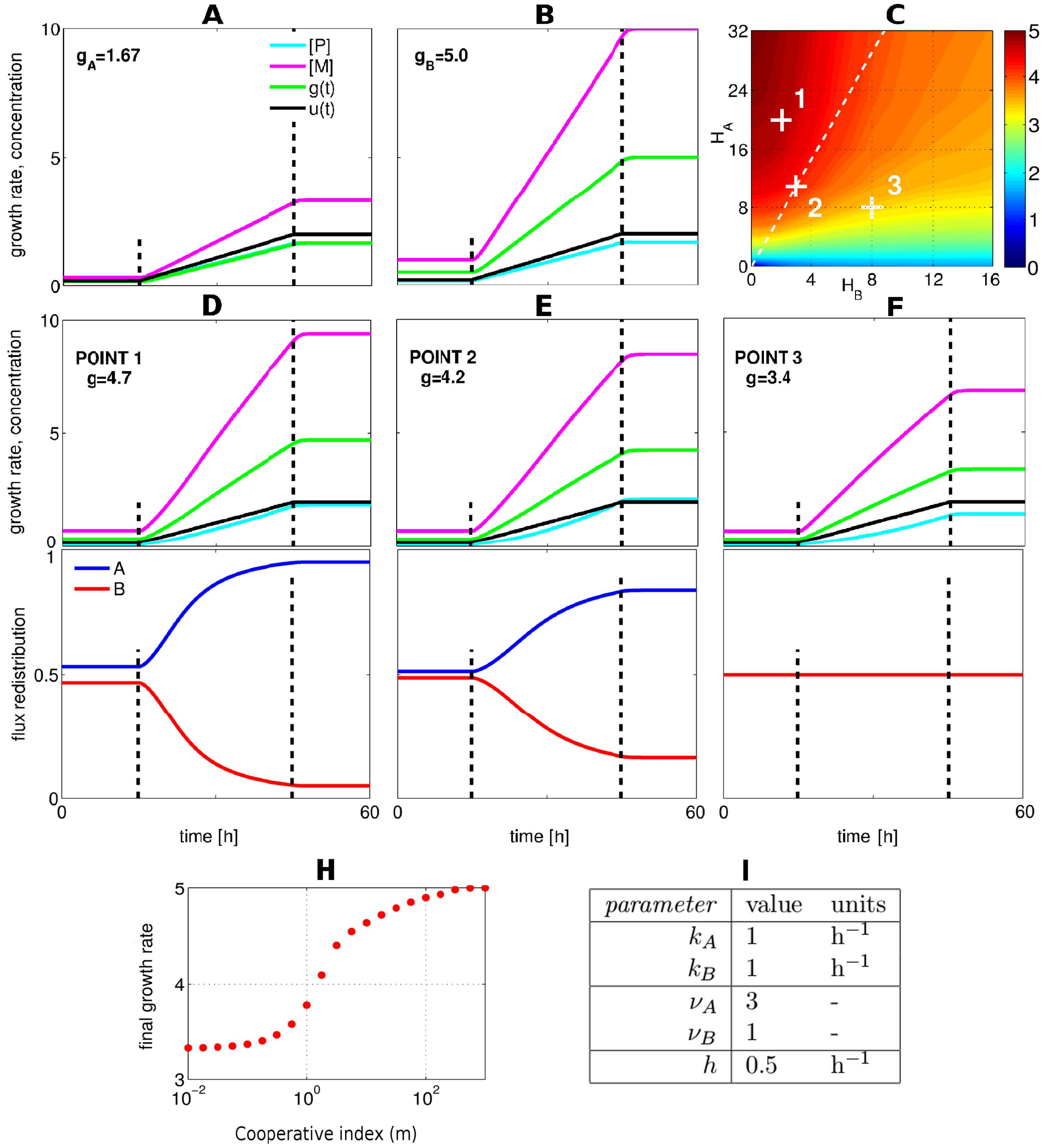

parameter	value	units
k_A	1	h^{-1}
k_B	1	h^{-1}
ν_A	3	-
ν_B	1	-
h	0.5	h^{-1}

Figure S10: **An example of implementation of greediness in a complex network with sub-optimal end-point. (A):** Sketch of the network and table of the parameter values used for the simulations of Eq. (S11). Since $\nu_A > \nu_B$, reaction A is more efficient than reaction B. **(B):** Effect on the final growth rate of the Hill constants H_A and H_B (all other parameter values as in panel A). It is interesting to note the opposite shape of this surface when compared with Fig. 5B from the main text: here greediness, i.e. $H_A > H_B$, does not lead to high final growth rates. **(C):** Examples of dynamics at different values of H_A and H_B. When $H_A > H_B$, the systems is adopting a greedy and myopic strategy (reaction B is silenced) that leads to a suboptimal point (low growth final rate). When $H_A < H_B$ the greedy strategy is no longer adopted (reaction A is silenced) and the final point is closer to the optimum.

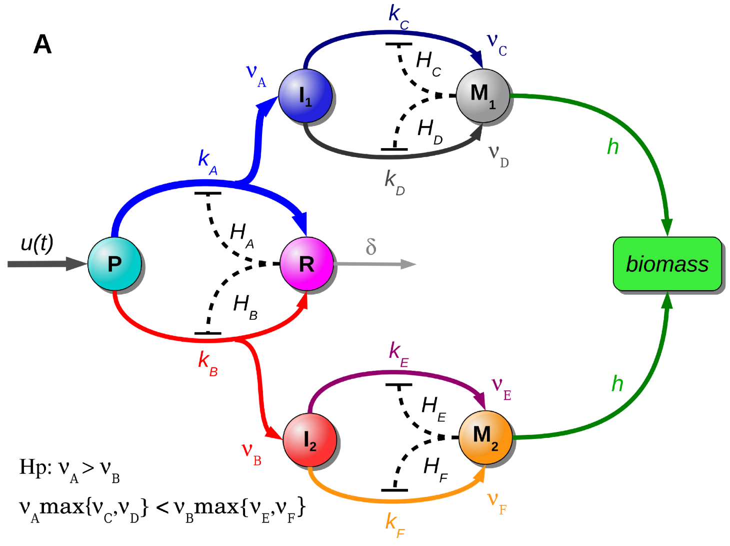

parameter	value	units
k_A	1	h^{-1}
k_B	1	h^{-1}
k_C	0.2	h^{-1}
k_D	0.2	h^{-1}
k_E	0.2	h^{-1}
k_F	0.2	h^{-1}
ν_A	4	-
ν_B	2	-
ν_C	1.5	-
ν_D	1	-
ν_E	4	-
ν_F	0.5	-
δ	0.5	h^{-1}
h	0.5	h^{-1}
H_C	8	h^{-1}
H_D	2	h^{-1}
H_E	8	h^{-1}
H_F	2	h^{-1}

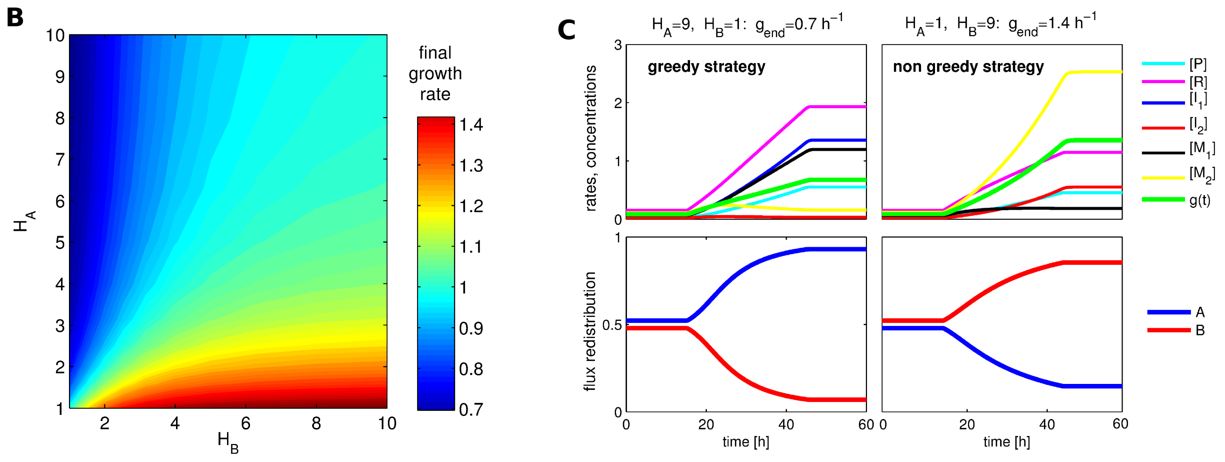

Figure S11: **Clustering of $d_c(t)$ trajectories.** **(A)**: Distance tree and the 3 resulting classes of $d_c(t)$, i.e. the distance of the trajectory to the straight line from $\mathbf{v}^{(0)}$ to $\mathbf{v}^{\text{opt}}$ (see Eq. (S12) in the SI text). **(B)**: Plots of the averaged trajectories of $d_c(t)$ grouped according to the clustering of panel (A). The grey color for AKG-*pck* indicates that this condition is borderline between classes I and II. **(C)**: Plots of $f(t)$: the persistent decrease of $f(t)$ indicates that the system constantly approaches the optimal point: often it is able to reach it ($f(t) \simeq 0$), sometimes it is trapped in a local minimum ($f(t) \gg 0$).

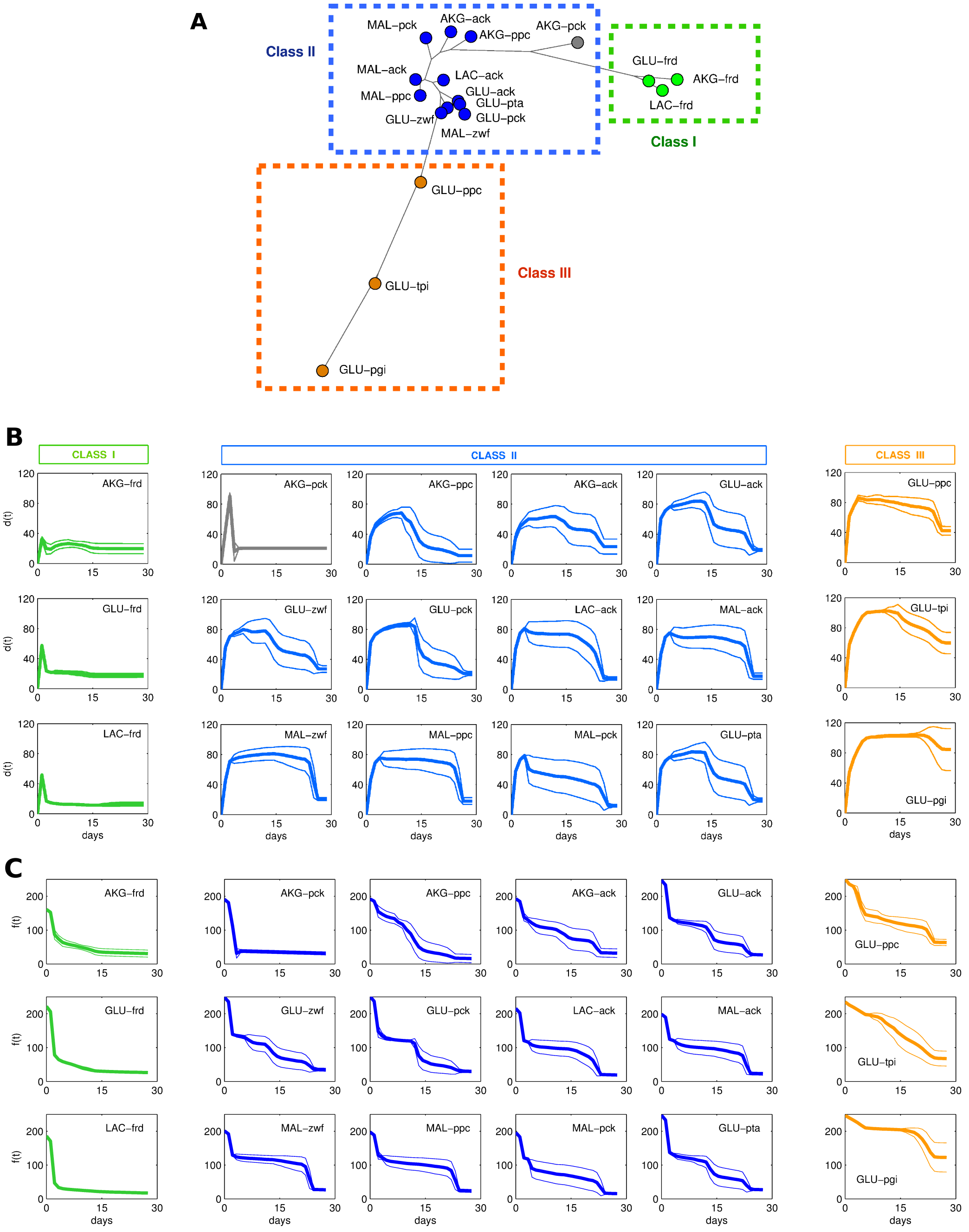

Figure S12: **Three examples of trajectories: LAC-*frd* (class I), GLU-*pck* (class II) and GLU-*tpi* (class III). (A-C):** The density plots show the time-recovery of the growth rate in our trajectories (color scale for the density: light color=low density; dark color=high density). The histogram in the right part of each panel reports the density of the growth rates at the end-point of the trajectories. In each panel one or two representative trajectories are shown explicitly. In the last column (*tpi* knockout on glucose) the brown line denotes a trajectory that reaches a high growth rate and the orange line one of low growth rate (see Fig. 4a-c in the maintext for more details). **(D-F):** Plot of the distance $d(t)$. Same visualization rules as in the first row. **(H-J):** Sketch of the trajectories in an hypothetical energy landscape (Hamiltonian $H = -g$) for the three classes. Black arrow denotes the shortest trajectory from $\mathbf{v}^{(0)}$ to $\mathbf{v}^{\text{opt}}$ while the "distance" here reported refers to the quantity $d(s)$, see Eq. (S12). Notice how in the GLU-*tpi* case the orange trajectory (low final growth rate) is trapped in a valley of local minimum.

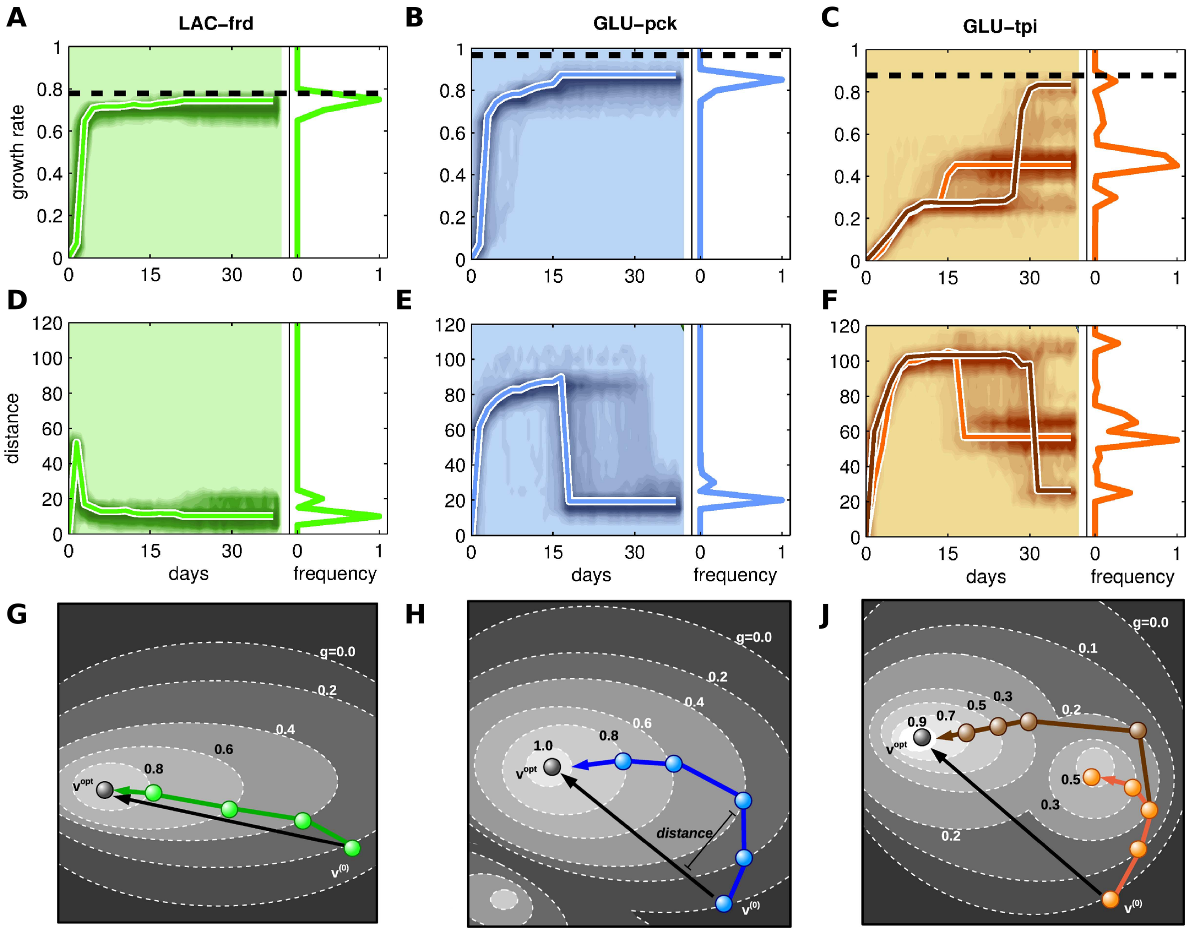

Table S1: **List of experimental conditions (carbon source and gene knockout pairs) considered in our validation.** The entry "$g(t)$" means that the dynamics of growth rate recovery have been compared with the experimental data. An entry with "^{13}C" indicates that a validation by ^{13}C-measured flux comparison has been carried out. With respect to the experiments reported in [6], ribose and glycerol have not been included as carbon sources because they are not present in the *E.coli* core metabolic network.

	Carbon Source				
Gene Knockout	α-ketoglutarate (AKG, $\triangle$)	glucose (GLU, $\circ$)	lactate (LAC, $\square$)	malate (MAL, $\Diamond$)	succinate (SUC, $\triangledown$)
acetate kinase (*ack*)	$g(t)$	$g(t)$	$g(t)$	$g(t)$	-
fumarate reductase (*frd*)	$g(t)$	$g(t)$	$g(t)$	-	-
glucose 6-phosphate dehydrogenase (*zwf*)	-	$g(t)$ and ^{13}C	-	$g(t)$	-
phophoenolpyruvate carboxylase (*ppc*)	$g(t)$	$g(t)$	-	$g(t)$	-
phophoenolpyruvate carboxykinase (*pck*)	$g(t)$	$g(t)$	-	$g(t)$	-
triose-phosphate isomerase (*tpi*)	-	$g(t)$ and ^{13}C	-	-	$g(t)$
phosphate transacetilase (*pta*)	-	^{13}C	-	-	-
phosphoglucose isomerase (*pgi*)	-	^{13}C	-	-	-
wild-type (*wt*)	$g(t)$	$g(t)$ and ^{13}C	$g(t)$	$g(t)$	$g(t)$

Table S2: **Comparison with some methods from the literature.** The table reports some statistics for describing the agreement between the ^{13}C-based experimental fluxes from [7, 4] and the predictions obtained by GRAM (reported in Fig. 3 in the main text), MOMA, ROOM, and pFBA. The comparison is performed using different statistical indexes, namely the Root Mean Square Deviation (RMSD), the correlation coefficient with the corresponding p-value and the cos-correlation coefficient defined as: $\rho_{\cos}(x, y) := \frac{x^T y}{||x|| \cdot ||y||}$, which still indicates a good correlation when close to 1. For GLU-*pgi*, values with * refer to the "low growth phenotype" whereas values with ** refer to the "high growth phenotype". Bold font denotes the best agreement.

	GRAM	MOMA	ROOM	pFBA	GRAM	MOMA	ROOM	pFBA
	GLU-*zwf*: intermediate steps							
RMSD	**0.1572**	0.3208	2.3476	0.5579	n.a.			
correlation	**0.9792**	0.8648	-0.0656	0.7114				
Log(p-value)	**-8.2370**	-3.8674	-0.0802	-2.1945				
cos-correlation	**0.9921**	0.94991	-0.2932	0.8557				
	GLU-*pta*: intermediate steps				GLU-*pta*: final steps			
RMSD	0.2014	**0.1667**	0.5148	0.3042	0.2225	0.3714	0.6016	**0.2171**
correlation	0.9868	**0.9934**	0.9281	0.9675	**0.9664**	0.8857	0.9328	0.9648
Log(p-value)	-14.7950	**-17.9997**	-7.4061	-11.0940	**-13.1365**	-7.7160	-10.0444	-12.9341
cos-correlation	0.9767	**0.9885**	0.8772	0.9469	**0.9778**	0.9333	0.9300	0.9776
	GLU-*ppc*: intermediate steps				GLU-*ppc*: final steps			
RMSD	**0.2385**	0.5438	0.4479	0.5262	**0.1469**	0.6947	0.6157	0.4090
correlation	**0.9576**	0.9051	0.9045	0.9041	**0.9791**	0.6660	0.8557	0.8598
Log(p-value)	**-9.2536**	-6.1483	-6.8536	-6.0024	**-15.2829**	-3.2823	-6.7102	-6.8352
cos-correlation	**0.9196**	0.8352	0.8604	0.8294	**0.9893**	0.8120	0.8923	0.9207
	GLU-*tpi*: intermediate steps				GLU-*tpi*: final steps			
RMSD	**0.4771**	0.6005	0.8334	1.1259	**0.3188**	0.7565	0.7340	1.0415
correlation	**0.7270**	0.6663	0.4228	0.2888	**0.8852**	0.4727	0.7298	0.4431
Log(p-value)	**-4.0706**	-3.2857	-1.3520	-0.7414	**-7.6959**	-1.6436	-4.1116	-1.4656
cos-correlation	**0.9000**	0.8582	0.7242	0.5700	**0.9505**	0.7736	0.8301	0.6449
	GLU-*pgi*: intermediate steps				GLU-*pgi*: final steps			
RMSD	**0.2062**	0.2483	0.8536	0.4897	**0.1461** *	0.2482*	0.7596*	0.3969*
					**0.2359****	0.3682**	0.7254**	0.3244**
correlation	**0.9470**	0.9400	0.5952	0.7681	**0.9776***	0.9507*	0.7276*	0.8595*
					**0.9429****	0.8856**	0.8197**	0.9148**
Log(p-value)	**-11.0977**	-10.5471	-2.5634	-4.7278	**-14.9626***	-11.4193*	-4.0782*	-6.8254*
					**-10.7682****	-7.7094**	-5.7693**	-8.9967**
cos-correlation	**0.9805**	0.9744	0.7344	0.8871	**0.9909***	0.9778*	0.8009*	0.9244*
					**0.9726****	0.9456**	0.8491**	0.9488**
